# RVFV virulence factor NSs triggers the mitochondrial MCL-1-BAK axis to activate pathogenic NLRP3 pyroptosis

**DOI:** 10.1371/journal.ppat.1012387

**Published:** 2024-08-30

**Authors:** Zhenqiong Guan, Huiling Li, Chongtao Zhang, Ziyan Huang, Meidi Ye, Yulan Zhang, Shufen Li, Ke Peng

**Affiliations:** 1 State Key Laboratory of Virology, Center for Antiviral Research, Wuhan Institute of Virology, Chinese Academy of Sciences, Wuhan, Hubei, China; 2 University of Chinese Academy of Sciences, Beijing, China; Loyola University Chicago, UNITED STATES OF AMERICA

## Abstract

Infection of Rift Valley fever virus (RVFV), a highly pathogenic mosquito-borne zoonotic virus, triggers severe inflammatory pathogenesis but the underlying mechanism of inflammation activation is currently unclear. Here, we report that the non-structural protein NSs of RVFV triggers mitochondrial damage to activate the NLRP3 inflammasome leading to viral pathogenesis in vivo. It is found that the host transcription inhibition effect of NSs causes rapid down-regulation of myeloid cell leukemia-1(MCL-1), a pro-survival member of the Bcl-2 (B-cell lymphoma protein 2) protein family. MCL-1 down-regulation led to BAK activation in the mitochondria, which triggered mtROS production and release of oxidized mitochondrial DNA (ox-mtDNA) into the cytosol. Cytosolic ox-mtDNA binds and activates the NLRP3 inflammasome triggering NLRP3-GSDMD pyroptosis in RVFV infected cells. A NSs mutant virus (RVFV-NSs^RM^) that is compromised in inducing transcription inhibition did not trigger MCL-1 down-regulation nor NLRP3-GSDMD pyroptosis. RVFV infection of the *Nlrp3*^*-/-*^ mouse model demonstrated that the RVFV-triggered NLRP3 pyroptosis contributed to RVFV inflammatory pathogenesis and fatal infection in vivo. Infection with the RVFV-NSs^RM^ mutant virus similarly showed alleviated inflammatory pathogenesis and reduced fatality rate. Taken together, these results revealed a mechanism by which a virulence factor activates the mitochondrial MCL-1-BAK axis through inducing host transcription inhibition to trigger NLRP3-dependent inflammatory pathogenesis.

## Author summary

RVFV is a highly pathogenic mosquito-borne zoonotic virus that infects both ruminants and humans. Humans infected by RVFV can develop acute hepatitis, encephalitis and hemorrhagic fever, which can result in fatal infection in severe cases. RVFV-triggered inflammation is an important driver for severe disease development but the underlying activation mechanism is currently unknown. Here, we found that the major virulence protein NSs of RVFV induces mitochondria damage in infected cells. This is achieved through the transcription inhibition effect of NSs which triggered transcriptional down-regulation of MCL-1 leading to mitochondrial BAK activation. Activated BAK then led to mitochondrial permeabilization and release of ox-mtDNA into the cytosol. The cytoplasmic ox-mtDNA binds and activates NLRP3 inflammasome leading to inflammatory pyroptosis. Using a RVFV infected mouse model and a mutant virus that did not trigger transcription inhibition, we showed that RVFV-triggered NLRP3 activation contributed to viral pathogenesis and fatal infection in vivo. In summary, we showed an important mechanism of RVFV triggered pathogenic inflammation, which may be employed by other viruses that induce transcription inhibition during infection.

## Introduction

Rift Valley fever virus (RVFV) is a highly pathogenic mosquito-borne zoonotic virus that infects both ruminants and humans [1,2]. RVFV causes severe disease in a range of wild and domestic ruminants, resulting in fetal malformation and abrupt abortion in 40 to 100% of pregnant ewes [3]. Newborn lambs are also highly susceptible to RVFV, and the mortality rate is estimated to be 95 to 100% [4]. Humans can be infected by RVFV by the bites of infected mosquitoes or through direct contact with bodily fluids from infected animals [1,3]. Humans infected by RVFV generally develop a self-limiting febrile illness, but in severe cases can develop diseases manifesting with acute hepatitis, encephalitis, hemorrhagic fever, which eventually can lead to fatal infection outcome [1,4]. Due to the potential of causing epidemic transmission and the lack of approved antiviral therapeutics for human use, RVFV is listed as a priority pathogen by the World Health Organization (WHO) [5]. Excessive inflammatory responses have been reported to be associated with RVFV pathogenesis in clinical patients and in infected animal models [6–9]. Revealing the mechanisms underlying RVFV triggered inflammation is important for understanding RVFV pathogenesis and for developing effective therapeutics against RVFV infection.

Being a member of the Phlebovirus genus in the Phenuiviridae family, RVFV contains a tripartite negative-sense, single stranded RNA genome, with the large (L), medium (M) and small (S) segments. The non-structural protein encoded by the S segment (NSs) of RVFV is the major virulence protein of the virus. Recombinant RVFV with deletion of NSs showed markedly reduced viral replication and pathogenesis in infected mouse model indicating its critical role in mediating viral pathogenesis in vivo [10–12]. A number of regulatory functionalities have been reported for NSs, including inhibition of host general transcription initiation, suppression of the IFN-β promoter and induction of proteasome-dependent down-regulation of dsRNA-dependent protein kinase (PKR) [13,14]. Whether the virulence factor NSs regulated RVFV-triggered inflammatory pathogenesis is currently unknown.

Mitochondria have recently emerged as important drivers of inflammation [15]. Mitochondria are surrounded by a double-membrane envelope composed of the mitochondrial outer membrane (MOM) and the mitochondrial inner membrane (MIM) [16]. The mitochondrial double-membrane compartments contain numerous constituents and metabolic products that can function as damage associated molecular patterns (DAMPs) to trigger inflammatory responses when released into the cytosol [15,16]. Release of these pro-inflammatory mitochondrial DAMPs (mtDAMPs), such as mitochondria DNA (mtDNA), are mediated through mitochondrial outer membrane permeabilization (MOMP) [17]. A major mechanism of MOMP formation involves activation of the proapoptotic pore-forming proteins BCL-2- associated X, apoptosis regulator (BAX) and BCL-2 antagonist/killer 1 (BAK) [18,19]. Upon activation, BAX and/or BAK form pores in the MOM that enable the extrusion of the MIM into the cytosol, culminating in MIM breakdown and release of mtDAMPs followed by activation of inflammatory responses [15]. Whether RVFV infection can trigger mitochondria-derived inflammation is currently unknown.

Here, we report that the NSs causes rapid transcriptional down-regulation of the BAK inhibitor myeloid cell leukemia-1(MCL-1), which led to BAK activation in the mitochondria. Activated BAK triggered mtROS production and release of oxidized mitochondrial DNA (ox-mtDNA) into the cytosol. Cytoplasmic ox-mtDNA binds and activates the NLRP3 inflammasome resulting in NLRP3-GSDMD pyroptosis in RVFV infected cells. A NSs mutant virus (RVFV-NSs^RM^) that is compromised in inducing transcription inhibition did not trigger MCL-1 down-regulation nor NLRP3-GSDMD pyroptosis. RVFV infection of the *Nlrp3*^*-/-*^ mouse model demonstrated that the RVFV-triggered NLRP3 pyroptosis contributed to RVFV inflammatory pathogenesis and fatal infection in vivo. Infection with the RVFV-NSs^RM^ mutant virus showed alleviated inflammatory pathogenesis and reduced fatality rate in the infected mouse model. Taken together, these results revealed a mechanism by which a virulence factor induces host transcription inhibition to trigger inflammatory pathogenesis through the MCL-1-BAK-NLRP3 axis.

## Results

### 1. RVFV infection triggers inflammatory pyroptosis

**Fig 1:**
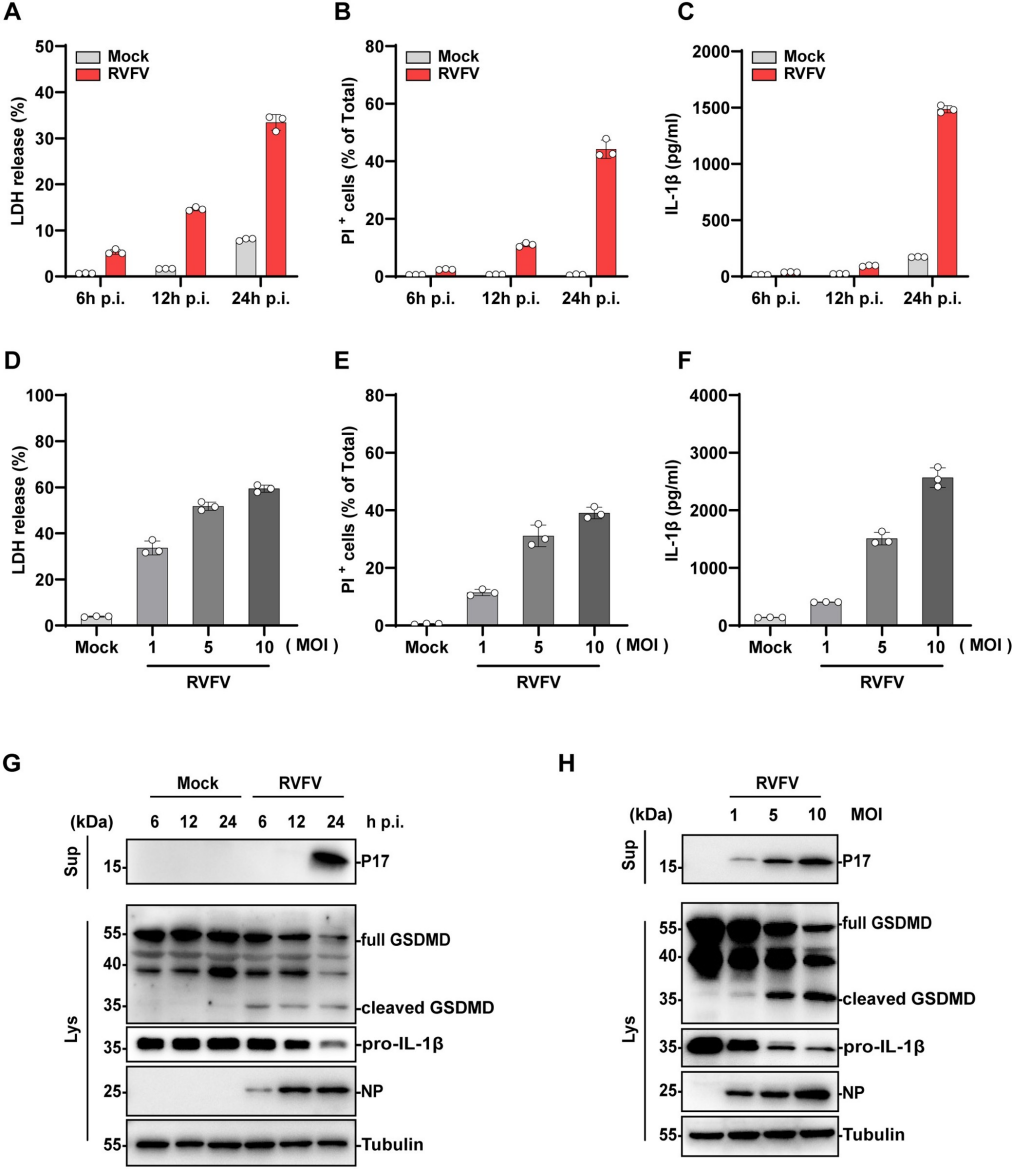
RVFV infection induces inflammatory cell death. (A -C) THP-1^PMA^ cells were infected with RVFV (MOI = 5) or mock treated. (A) LDH release and (B) PI uptake were quantified at 6,12, 24 h p.i. (C) Mature IL-1β in the supernatant were determined by ELSA at indicated time points. (D-F) THP-1^PMA^ cells were infected with RVFV with indicated MOI for 24 h. (D) LDH release, (E) PI uptake, (F) mature IL-1β in the supernatant were determined as above.(G) Immunoblot analysis of mature IL-1β (P17) in supernatants (Sup) and cleaved GSDMD in cell lysates (Lys) of THP-1^PMA^ cells infected with RVFV (MOI = 5) with indicated time.(H) Immunoblot analysis of mature IL-1β (P17) in supernatants (Sup) and cleaved GSDMD in cell lysates (Lys) of THP-1^PMA^ cells infected with RVFV with indicated MOI at 24 h p.i.. Data are shown as mean ± SD from three independent experiments. Immunoblot results are representative of three independent experiments.

First we analyzed whether RVFV infection induced inflammatory cell death. Macrophages are an important cell type that mediates RVFV infection and pathogenesis in vivo [1,8,20,21]. We therefore used RVFV infected phorbol 12-myristate 13-acetate (PMA)-differentiated THP-1-derived macrophages (THP-1^PMA^) as a model for this analysis. THP-1^PMA^ cells were infected with RVFV and cell lysates and supernatants were harvested for analyzing cell death activation. RVFV infection led to a significant increase in lactate dehydrogenase (LDH) release into the supernatant (Fig 1A) and accumulation of intracellular propidium iodide (PI) staining (Figs 1B and S1A) over time. ELISA analysis showed that RVFV infection triggered IL-1β release into the supernatant (Fig 1C), indicating the activation of inflammatory cell death. Infection of THP-1^PMA^ cells with serial MOIs of RVFV led to release of LDH, accumulation of intracellular PI staining, and IL-1β release in a dose-dependent manner (Figs 1D–1F and S1B). These results showed that RVFV infection triggers inflammatory cell death activation with pro-inflammatory cytokine IL-1β release.

To characterize the mode of inflammatory cell death triggered by RVFV infection, we examined key molecules associated with different inflammatory cell death pathways. Western blot (WB) analysis revealed the cleavage of active gasdermin D (GSDMD) in RVFV infected cells concomitantly with mature IL-1β (P17) release into the supernatant, suggesting the activation of pyroptosis (Fig 1G). Activation of pyroptosis was observed in a dose-dependent manner in cells infected with serial MOIs of RVFV (Fig 1H). We also analyzed whether RVFV triggered necroptosis, another inflammatory cell death pathway that is often triggered during virus infection [22–24]. In contrast to activation of pyroptosis, the phosphorylation of MLKL (pMLKL), marker of necroptosis activation, was not detected in RVFV-infected cells (S1C and S1D Fig). In contrast, cells treated with a combination of lipopolysaccharide (LPS), SM-164, and z-VAD (L/S/Z) induced distinct detection of pMLKL, serving as the positive control (S1C and S1D Fig). This indicated that RVFV infection did not trigger necroptosis in this cell model. Collectively, these results suggested that RVFV triggers inflammatory pyroptosis in infected cells.

### 2. RVFV infection triggers NLRP3 inflammasome activation

**Fig 2:**
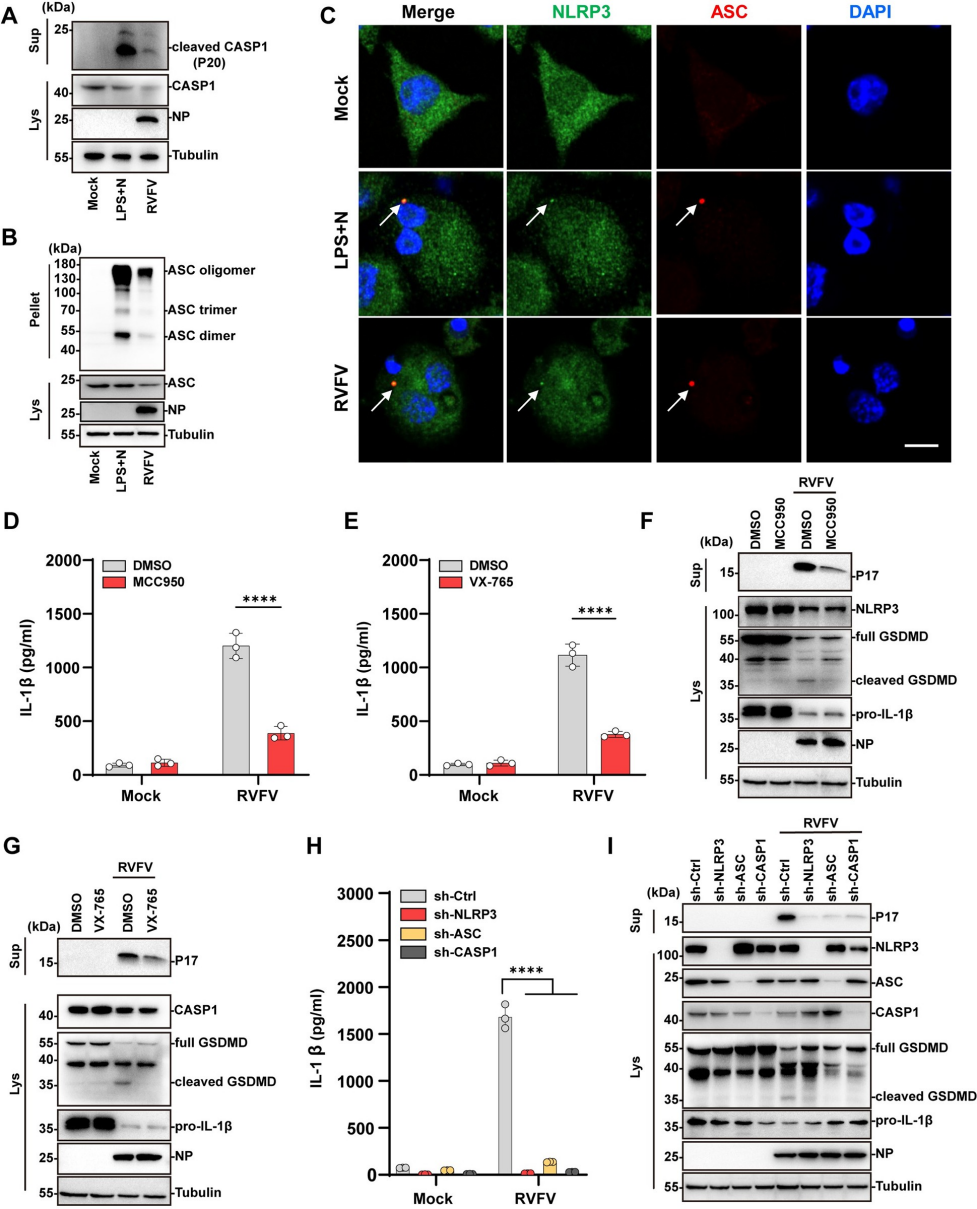
RVFV infection activates NLRP3 inflammasome. (A) Immunoblot analysis of cleaved CASP1 in the supernatant of THP-1^PMA^ cells treated with LPS and nigericin (LPS + N), or infected with RVFV (MOI = 5) for 24 h. (B) Immunoblot analysis of ASC oligomerization in THP-1^PMA^ cells treated with LPS and nigericin, or infected with RVFV (MOI = 5) for 12h. (C) THP-1^PMA^ cells were treated with LPS and nigericin or infected with RVFV (MOI = 5) for 12h. Cells were co-stained for NLRP3 (green), ASC (red), and DAPI (blue). Arrow indicates co-localization of NLRP3 and ASC signals. Scale bar,10 μm. (D) THP-1^PMA^ cells were pre-treated with NLRP3 inhibitor MCC950 (20 μM) followed by RVFV (MOI = 5) infection for 24h. IL-1β release was quantified by ELISA. (E) THP-1^PMA^ cells were pre-treated with caspase1 inhibitor VX-765(20 μM) followed by RVFV (MOI = 5) infection for 24 h. IL-1β release was quantified by ELISA. (F) Immunoblot analysis of cleaved GSDMD in RVFV-infected THP-1^PMA^ cells pre-treated with MCC950 or DMSO. (G) Immunoblot analysis of cleaved GSDMD in RVFV-infected THP-1^PMA^ cells pre-treated with VX-765 or DMSO. (H) THP-1^PMA^ cells stably expressing non-targeting shRNA (sh-Ctrl) or specific shRNA against NLRP3, ASC, CASP1 (sh-NLRP3, sh-ASC, sh-CASP1) were infected with RVFV (MOI = 5) for 24h. IL-1β release were quantified by ELISA. (I) Immunoblot analysis of cleaved GSDMD in RVFV-infected THP-1^PMA^ cells stably expressing non-targeting shRNA (sh-Ctrl) or specific shRNA against NLRP3, ASC, CASP1(sh-NLRP3, sh-ASC, sh-CASP1). Data are shown as mean ± SD from three independent experiments. Statistical significance was analyzed by two-way ANOVA. ****p < 0.0001. Immunoblot results are representative of three independent experiments.

Next, we analyzed the underlying mechanisms of RVFV triggered pyroptosis. RVFV infection of THP-1^PMA^ cells led to the release of cleaved caspase-1(CASP1) maturation fragment (p20) and the oligomerization of apoptosis-associated speck-like protein containing a CARD (ASC), similar with the lipopolysaccharide (LPS)- and nigericin-treated cells which were included as a control (Fig 2A and 2B). Immunofluorescence analysis revealed a distinct co-localization between ASC and NLRP3 specks in the cytoplasm of RVFV infected cells, indicating the involvement of NLRP3 inflammasome (Fig 2C). ELISA analysis showed that treatment with MCC950 (an NLRP3 specific inhibitor) or VX-765 (a CASP1 specific inhibitor) significantly inhibited IL-1β release into the supernatant from RVFV infected cells (Fig 2D and 2E). Western blot analysis of cell lysates from RVFV infected cells showed that both MCC950 and VX-765 treatment inhibited GSDMD cleavage concomitantly with reduced IL-1β release (Fig 2F and 2G). To further verify the role of NLRP3 inflammasome in RVFV-induced pyroptosis, we depleted NLRP3, ASC and CASP1 respectively in THP-1^PMA^ cells with lentivirus mediated gene silencing and then performed RVFV infection. Similar to the VX765 or MCC950 treatment, RVFV induced IL-1β release and GSDMD cleavage were reduced in NLRP3, ASC or CASP1 depleted cells (Fig 2H and 2I). Since NLRP3-dependent pyroptosis is executed by GSDMD, we also depleted GSDMD to verify its role in RVFV-infection induced IL-1β release. In line with the above data, mature IL-1β (P17) and LDH released into the supernatants were reduced in RVFV infected GSDMD depleted cells compared with the infected control cells (S2A–S2C Fig). Taken together, these results suggested that RVFV infection activates the NLRP3 inflammasome to trigger pyroptosis.

### 3. RVFV infection triggers mtDNA release to activate the NLRP3 inflammasome

**Fig 3:**
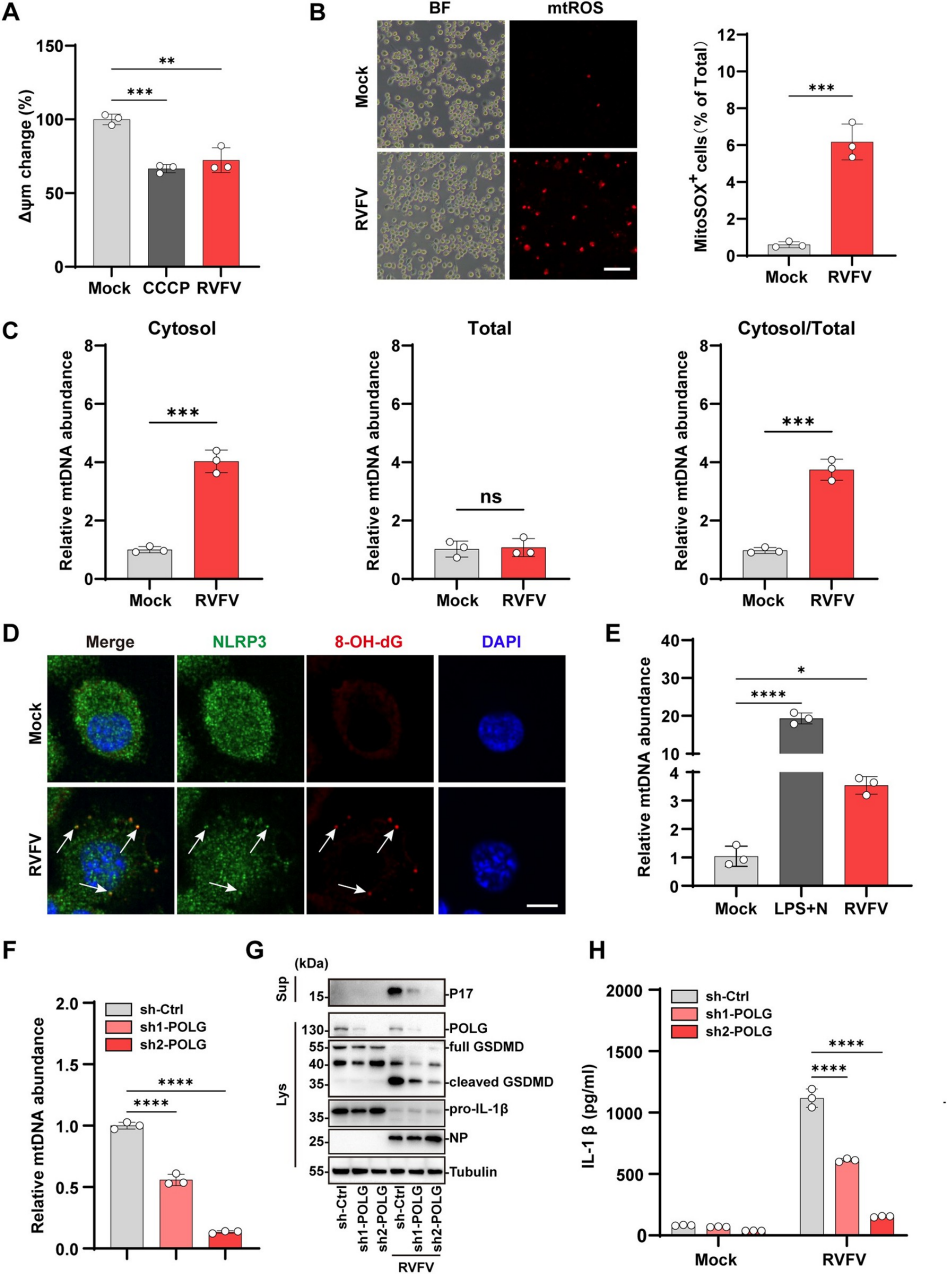
RVFV infection induces mtDNA release to activate NLRP3 inflammasome. (A) THP-1^PMA^ cells were treated with CCCP (50 μM) for 3h or infected with RVFV (MOI = 5) for 12 h. Changes in mitochondrial outer membrane potential (△ψ_m_) were measured by staining with TMRM probe. (B) THP-1^PMA^ cells were infected with RVFV (MOI = 5) for 12 h. Intracellular mtROS was stained with MitoSOX (scale bar, 100 μm). (C) THP-1^PMA^ cells infected with RVFV (MOI = 5) for 12 h were collected and total or cytosolic DNA was extracted. Total or cytosolic mtDNA levels were determined with qPCR and normalized to mock infected controls. (D) THP-1^PMA^ cells were infected with RVFV (MOI = 5) for 12 h. Cells were co-stained for NLRP3 (green), 8-OH-dG (red), and DAPI (blue). Arrow indicates co-localization of NLRP3 and 8-OH-dG signals. Scale bar, 10μm. (E) THP-1^PMA^ cells were infected with RVFV (MOI = 5) for 12 h. mtDNA bound to NLRP3 was determined via immunoprecipitation of NLRP3 followed by qPCR analysis of mtDNA. (F) Intracellular total level of mtDNA in THP-1 cells stably expressing non-targeting control shRNA or shRNAs against POLG was detected by qPCR. (G and H) THP-1^PMA^ cells stably expressing non-targeting shRNA (sh-Ctrl) or shRNAs against POLG were infected with RVFV (MOI = 5) for 24 h.(G) Immunoblot analysis of P17 level in supernatants and POLG, activated GSDMD in cell lysates. (H) IL-1β release were determined by ELISA. Data are shown as mean ± SD from three independent experiments. Statistical significance was analyzed by one-way ANOVA in (A, E, and F), or Student’s t-test in (B) and (C), or two-way ANOVA in (H). **p < 0.01; ***p < 0.001; ****p < 0.0001; ns, no significance. Immunoblot results are representative of three independent experiments.

Next, we investigated the underlying mechanisms of RVFV triggered NLRP3 activation. Recent studies revealed that mitochondria damage serves as an important driver to activate pyroptosis [15,25]. We therefore analyzed whether RVFV infection induces mitochondria damage. Carbonyl cyanide 3-chlorophenylhydrazone (CCCP) is an oxidative phosphorylation uncoupler that can induce the opening of the permeability transition pore on the mitochondrial membrane leading to the dissipation of mitochondrial outer membrane potential (△ψ_m_) and mitochondrial damage [26]. In RVFV-infected THP-1^PMA^ cells, a reduction in △ψ_m_ was observed as indicated by the reduced tetramethyl rhodamine methyl ester (TMRM) staining, similar with CCCP treatment which was included as a control (Fig 3A). In the case of mitochondrial disorders, lower △ψ_m_ was observed to stimulate accumulation of mitochondrial reactive oxygen species (mtROS) [27,28]. Indeed, RVFV infection also led to pronounced accumulation of mtROS as indicated by the MitoSOX staining in RVFV-infected cells (Fig 3B). These data suggested that RVFV infection triggers mitochondrial damage.

Damaged mitochondria can release molecular DAMPs including mtDNA to trigger pyroptosis [15,29,30]. Using quantitative PCR (qPCR) analysis, we detected a significant increase in cytosolic mtDNA copy numbers in the RVFV infected-cells compared to the mock infected control cells (Fig 3C). Quantification analysis showed that RVFV infection did not affect the total amount of intracellular mtDNA (Fig 3C, middle panel) confirming that RVFV infection triggers cytosolic release of mtDNA from damage mitochondria. Recent evidences suggest that oxidized mtDNA (ox-mtDNA) serves as a binding ligand to trigger NLRP3 inflammasome activation [31–33]. In RVFV infected cells, mtDNA could be oxidized by the mtROS during its cytosolic release. Indeed, labeling with 8-OH-dG antibody, which recognized oxidized DNA, demonstrated accumulation of ox-mtDNA in the cytosol of RVFV infected cells (Fig 3D). Immunofluorescence microscopy analysis further showed distinctive co-localization between the ox-mtDNA and the NLRP3 specks (Fig 3D). The NLRP3 antibody precipitated significantly more mtDNA in RVFV infected cells than in mock infected cells, in which the LPS and nigericin treatment was included as a control (Fig 3E). These results together indicated that RVFV triggered oxidized mtDNA accumulation in the cytosol which binds and activates the NLRP3 inflammasome.

To further verify the role of mtDNA in triggering NLRP3 inflammasome activation during RVFV infection, we depleted DNA polymerase gamma (POLG), which is responsible for mitochondrial DNA replication [34]. Depletion of POLG led to a significant reduction of total intracellular mtDNA abundance (Fig 3F). Of the two shRNAs targeting POLG, sh2 showed a more effective depletion of POLG (Fig 3G). Correspondingly, reduction of total intracellular mtDNA level was more pronounced in POLG-sh2 transduced cells than in POLG-sh1 transduced cells (Fig 3F). POLG depletion reduced RVFV induced IL-1β release and GSDMD cleavage compared with the infected control cells (Fig 3G and 3H). Notably, in all these above measurements of RVFV infected cells, POLG-sh2 transduced cells showed more pronounced reduction or inhibition than those in the POLG-sh1 transduced cells, reflecting the role of mtDNA abundance in triggering these responses. Taken together, these results suggested that RVFV infection induced oxidized mtDNA release to activate the NLRP3 inflammasome.

### 4. BAK mediates RVFV triggered mtDNA release and NLRP3 activation

**Fig 4:**
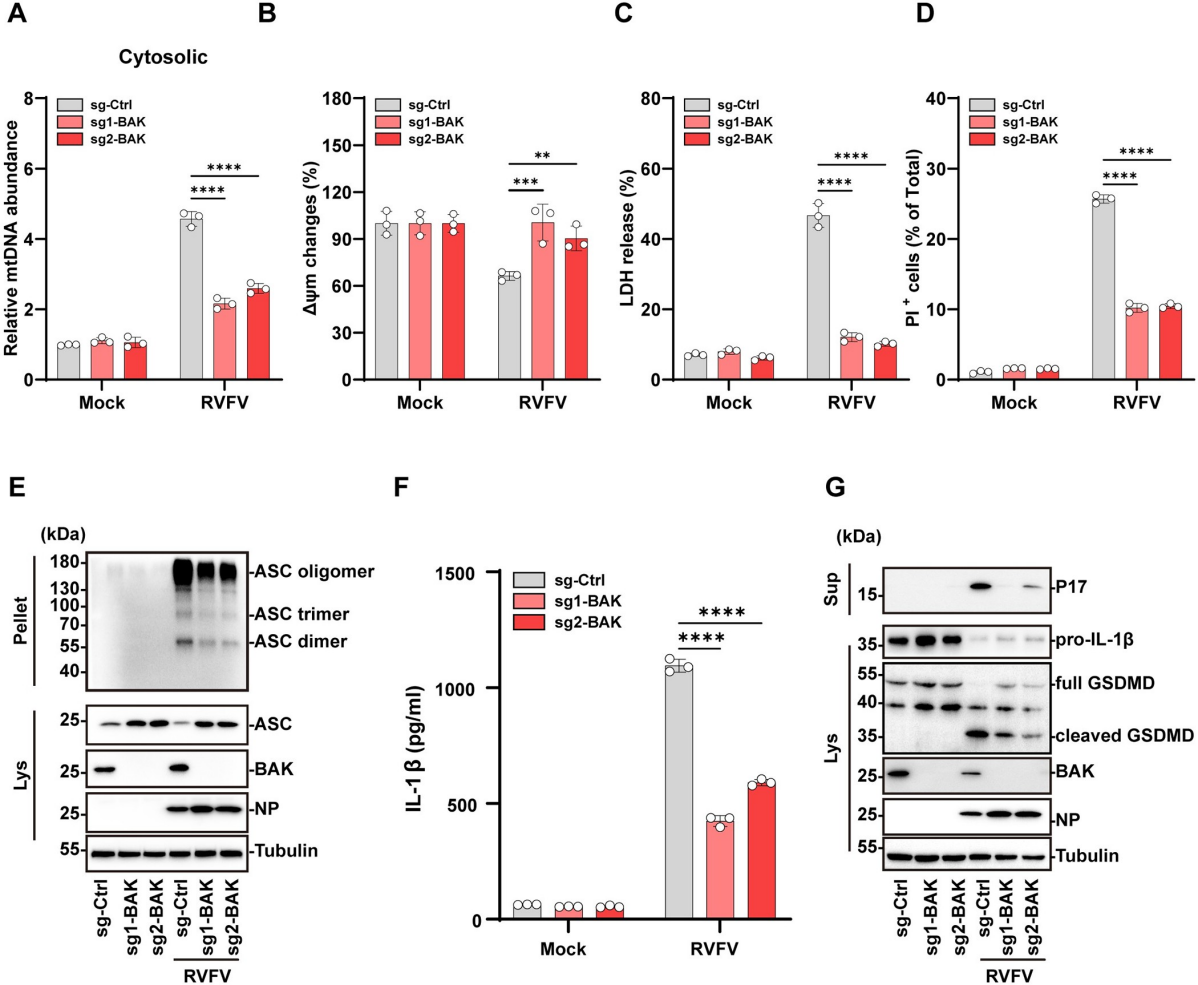
BAK mediates RVFV-induced pyroptosis. (A-G) THP-1 cells stably expressing non-targeting sgRNA (sg-Ctrl) or sgRNAs against BAK were infected with RVFV (MOI = 5) for indicated time. (A) Cytosolic mtDNA levels were determined with qPCR at 12h p.i.. (B) Changes in mitochondrial outer membrane potential (△ψ_m_) were measured at 12 h p.i.. (C) LDH release was quantified at 24 h p.i.. (D) PI uptake was quantified at 18 h p.i.. (E) Immunoblot analysis of ASC oligomerization at 12 h p.i.. (F) IL-β release in supernatants was quantified by ELISA at 24 h p.i.. (G) Immunoblot analysis of P17 level in supernatants, and pro-IL-1β, cleaved GSDMD, BAK expression in cell lysates at 24 h p.i.. Data are shown as mean ± SD from three independent experiments. Statistical significance analysis was analyzed by two-way ANOVA. **p < 0.01; ***p < 0.001; ****p < 0.0001. Immunoblot results are representative of three independent experiments.

We next investigated the underlying mechanism of RVFV-induced mtDNA release. As a mitochondrial DAMP, mtDNA release occurs upon MOMP formation which can be resulted from BAK or BAX activation [15,16]. Immunofluorescence analysis showed that BAK formed distinctive aggregation in mitochondria in RVFV-infected cells similar with STP treatment, which was included as a control to induce MOMP formation (S3A Fig). Therefore, we investigated whether BAK or BAX mediated RVFV-induced mtDNA release. For this purpose, BAK or BAX was depleted in cells, followed by RVFV infection and quantification of the cytosolic mtDNA. As shown in Fig 4A, BAK depletion significantly reduced release of cytosolic mtDNA in RVFV infected cells compared with the infected control cells. In contrast, BAX depletion did not affect RVFV triggered release of cytosolic mtDNA (S3B Fig). In addition, RVFV-induced reduction of mitochondrial membrane potential was rescued in BAK-deficient cells (Fig 4B). BAK depletion also reduced RVFV triggered cell death as indicated by lower LDH release and decreased PI uptake compared with infected control cells (Fig 4C and 4D). In contrast, BAX depletion did not inhibit RVFV induced LDH release compared with the infected control cells (S3C Fig). Furthermore, depletion of BAK reduced RVFV-induced ASC oligomerization, IL-1β release, and GSDMD cleavage (Fig 4E–4G). Differently, depletion of BAX did not inhibit RVFV-induced IL-1β release or GSDMD cleavage (S3D and S3E Fig). These results indicated that BAK, but not BAX, plays a critical role in RVFV induced mtDNA release and NLRP3 inflammasome activation.

### 5. RVFV NSs induces down-regulation of MCL-1 to activate BAK

**Fig 5:**
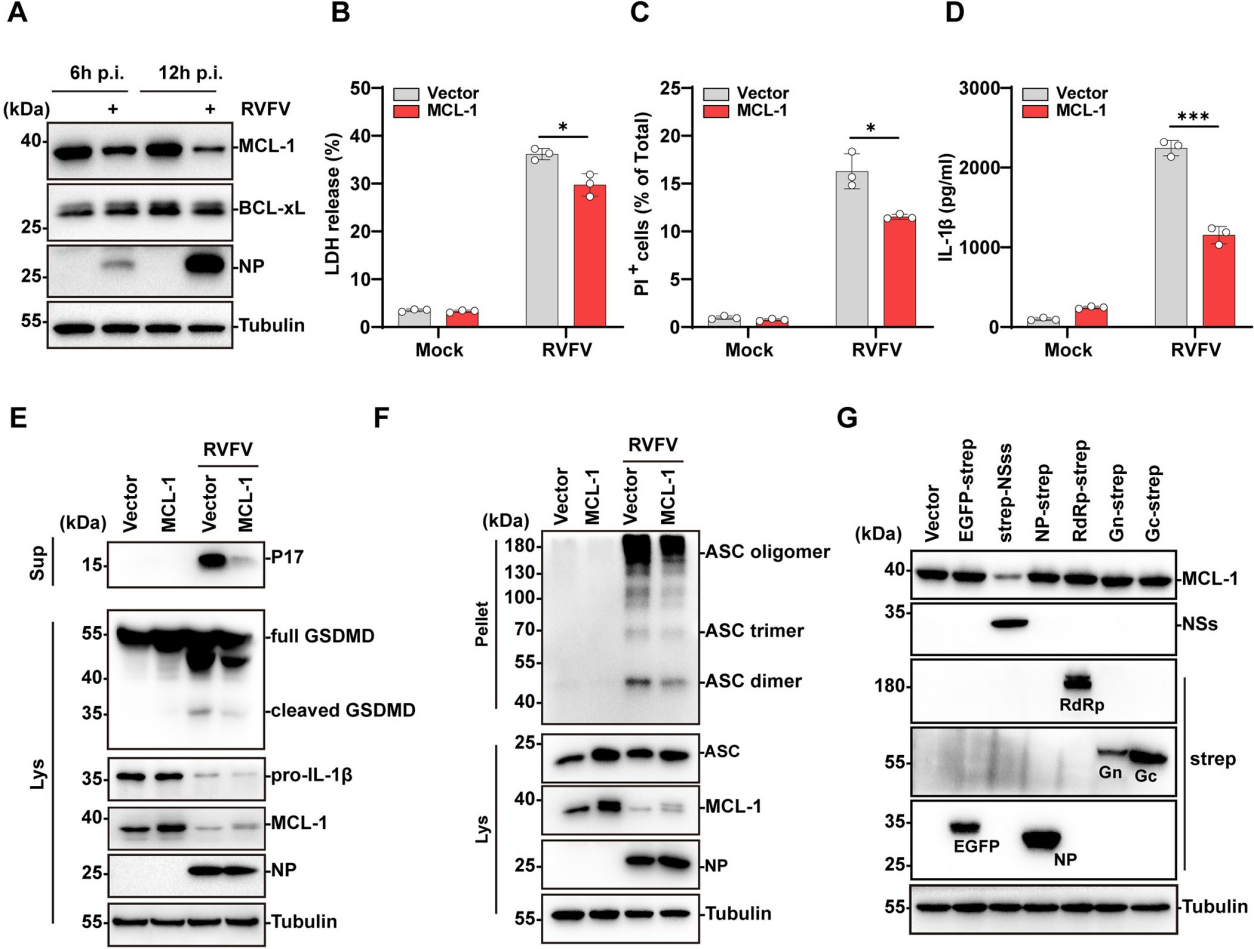
RVFV NSs induced MCL-1 downregulation is required to activate BAK. (A) Immunoblot analysis of MCL-1 expression in mock-infected or RVFV-infected THP-1^PMA^ cells at 6,12 h p.i.. (B-F) THP-1 cells transduced with lentiviruses expressing vector or Flag-MCL-1 and infected with RVFV (MOI = 5) for 18 h in (B-E) or 12 h in (F). (B) LDH release was quantified. (C) PI uptake was quantified. (D) IL-1β release in supernatants was quantified by ELISA. (E) Immunoblot analysis of P17 level in supernatants, and cleaved GSDMD, MCL-1 expression in cell lysates. (F) Immunoblot analysis of ASC oligomerization. (G) HEK293T cells were transfected with plasmids expressing viral proteins. After 24 hours post transfection, the cell lysates were used for immunoblot analysis of MCL-1 and viral protein expression. Data are shown as mean ± SD from three independent experiments. Statistical significance was analyzed by two-way ANOVA. * p < 0.05; ***p < 0.001. Immunoblot results are representative of three independent experiments.

Next, we investigated the mechanisms that drive BAK activation during RVFV infection. Under homeostatic conditions, BAK is sequestered and inhibited by the pro-survival BCL-2 family members MCL-1 and BCL-xL to block its pro-apoptotic activity [35,36]. WB analysis of RVFV infected cell lysates showed that MCL-1 was rapidly down-regulated in RVFV-infected cells 6 hours post infection while the abundance of BCL-xL was not affected (Fig 5A). This suggested that MCL-1 is sensitive to RVFV infection and may contribute to RVFV triggered BAK activation. To verify whether MCL-1 down-regulation triggered BAK mediated inflammation during RVFV infection, we over-expressed MCL-1 in THP-1^PMA^ cells using lentivirus mediated transduction. Overexpressing MCL-1 in cells alleviated RVFV triggered cell death as indicated by reduced LDH release (Fig 5B) and reduced intracellular PI uptake (Fig 5C). Overexpression of MCL-1 also significantly reduced IL-1β release in RVFV infected THP-1^PMA^ cells (Fig 5D). Furthermore, GSDMD cleavage was reduced in RVFV-infected cells overexpressing MCL-1, indicating of reduced pyroptosis (Fig 5E). Consistent with alleviated inflammasome activation, ASC oligomerization was reduced in RVFV-infected cells overexpressing MCL-1 compared with the infected control cells (Fig 5F). It was observed that the overexpressed MCL-1 was also efficiently degraded in RVFV infected cells, despite being maintained at a higher level than in the cells transduced by the empty vector (Fig 5E and 5F). The reduction of overexpressed MCL-1 may explain the partial inhibition effects observed in the above experiments. Nevertheless, these results suggested that intracellular MCL-1 abundance is important in inhibiting RVFV-induced pyroptosis.

Next, we investigated the mechanisms underlying RVFV induced MCL-1 down-regulation. For this purpose, virus proteins including NP, NSs, RdRp, Gn, Gc were transiently expressed in HEK293T cells through transfection and the MCL-1 protein abundance was analyzed by western blot analysis. It was found that expression of NSs but not the other viral proteins led to pronounced MCL-1 down-regulation (Fig 5G). To verify the role of NSs in mediating MCL-1 down-regulation during RVFV infection, a NSs deleted RVFV mutant virus, RVFV^△NSs^, was constructed based on the reverse genetic system [10]. Consistent with the notion that NSs mediates MCL-1 down-regulation, MCL-1 abundance in RVFV^△NSs^-infected cells was comparable with the mock infected control cells, while infection with wild-type RVFV led to distinct MCL-1 down-regulation (S4A Fig). LDH release was also reduced in RVFV^△NSs^-infected cells compared with the wild-type RVFV infected cells (S4B Fig). These results suggested that NSs mediated RVFV-induced MCL-1 down-regulation.

### 6. RVFV NSs induces down-regulation of MCL-1 through transcription inhibition

**Fig 6:**
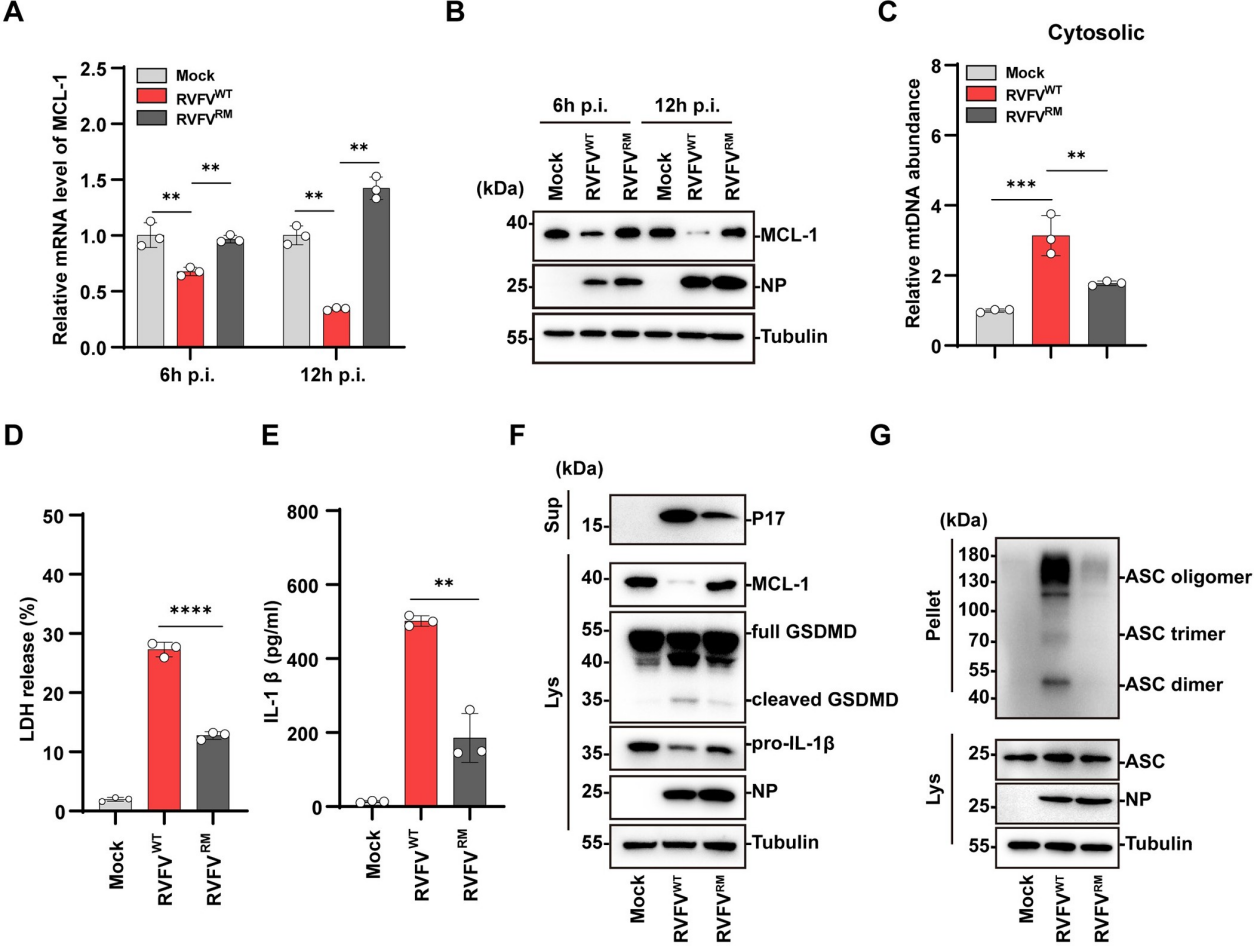
RVFV NSs induces transcriptional down-regulation of MCL-1 to promote pyroptosis. (A-G) THP-1^PMA^ cells were mock-infected or wild type RVFV-infected or RVFV-NSs^RM^ (RVFV^RM^)-infected (MOI = 5) at indicated time. (A) mRNA level of MCL-1 was measured by qRT-PCR. (B) Immunoblot analysis of MCL-1, NP in cell lysates. (C) The cytosolic level of mtDNA was quantified by qPCR. (D) LDH release was quantified at 24 h p.i.. (E) IL-1β release in supernatants was quantified by ELISA at 24 h p.i.. (F) Immunoblot analysis of P17 level in supernatants, and cleaved GSDMD, MCL-1, NP expression in cell lysates at 24 h p.i.. (G) Immunoblot analysis of ASC oligomerization at 12 h p.i.. Data are shown as mean ± SD from three independent experiments. Statistical significance was analyzed by Student’s t-test. **p < 0.01; ***p < 0.001; ****p < 0.0001. Immunoblot results are representative of three independent experiments.

Next, we investigated the underlying mechanisms of NSs triggered MCL-1 down-regulation. RT-qPCR showed that the mRNA level of MCL-1 was significantly down-regulated in wild-type RVFV-infected but not in RVFV^△NSs^ virus infected cells 6 h p.i., indicating down-regulation of MCL-1 at the RNA level (S5A Fig). A major virulence mechanism of NSs is suppression of the host transcription initiation leading to down-regulation of host mRNA transcripts [37,38]. We therefore investigated whether NSs induced down-regulation of MCL-1 depends on its transcription inhibition effects. A previous study identified a dual-mutation on NSs, the R16H and M250K mutation, which impaired NSs functionality in suppressing the host general transcription initiation [39]. To investigate whether NSs triggers MCL-1 RNA down-regulation through transcription inhibition, we introduced the R16H and M250K mutations into the recombinant RVFV-NSs^RM^ virus using the reverse genetics system. THP-1^PMA^ cells were infected with RVFV-NSs^WT^ or RVFV-NSs^RM^ virus and the intracellular level of MCL-1 was analyzed. It is found that the RVFV-NSs^WT^ virus triggered pronounced reduction of MCL-1 both at the RNA and protein level, while the RNA or protein level of MCL-1 was not reduced in RVFV-NSs^RM^ -infected cells (Fig 6A and 6B). RVFV-NSs^RM^-infected THP-1^PMA^ cells also showed reduction in the release of mtDNA into the cytosol, and LDH, IL-1β into the supernatant compared with the RVFV-NSs^WT^ virus infected cells (Fig 6C–6E). In addition, the cleavage of GSDMD and ASC oligomerization were reduced in RVFV-NSs^RM^-infected cells compared with the RVFV-NSs^WT^ virus infected cells (Fig 6F and 6G). Reduced accumulation of mtROS was also observed in RVFV-NSs^RM^-infected cells in line with reduced mitochondrial damage triggered by the RVFV-NSs^RM^ virus (S5B Fig). Collectively, these results indicated that RVFV NSs protein triggers down-regulation of MCL-1 through inducing host transcription inhibition, which promoted BAK activation and triggered downstream pyroptosis.

### 7. RVFV triggered NLRP3 pyroptosis contributes to viral pathogenesis in vivo

**Fig 7:**
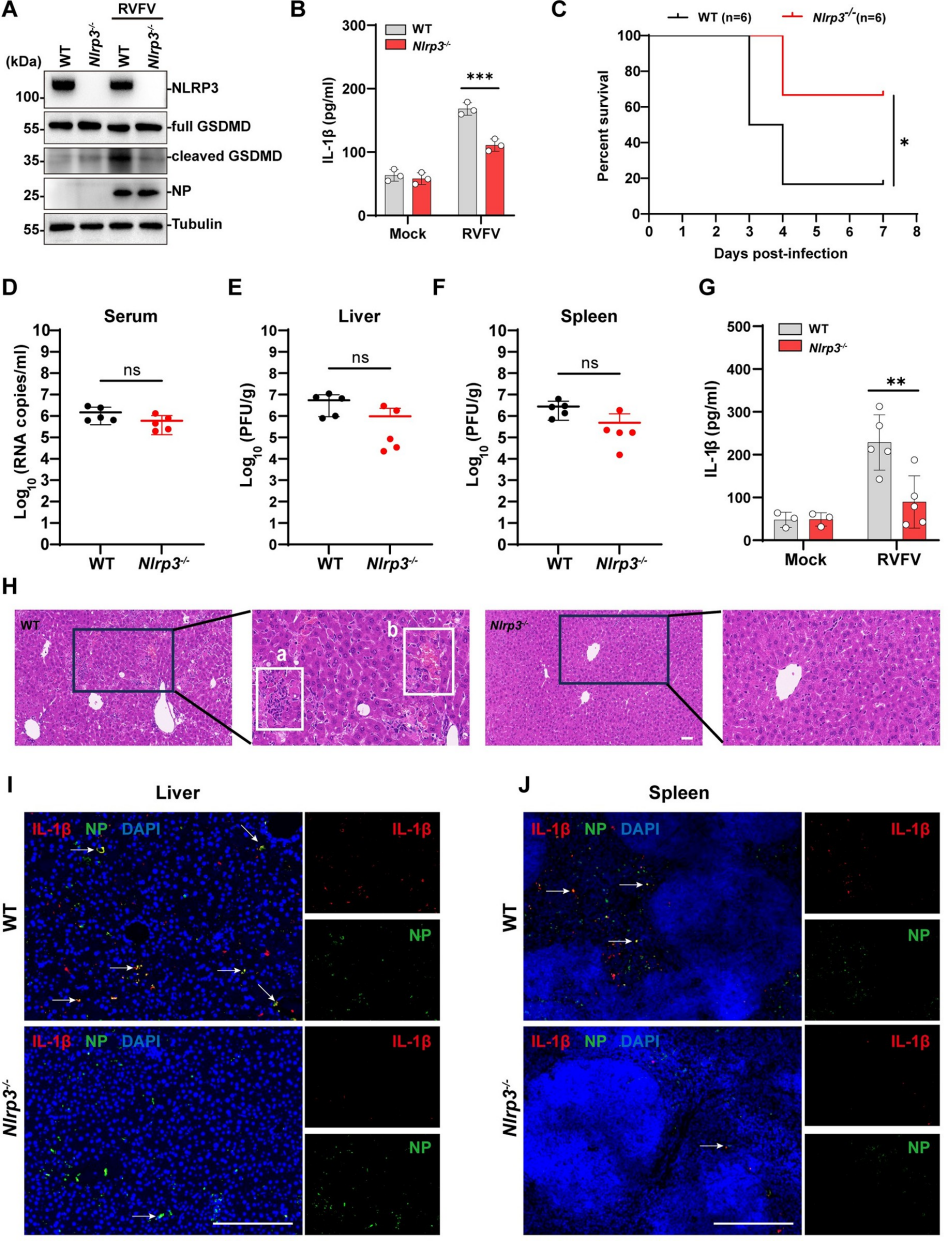
RVFV triggered NLRP3 activation promotes viral pathogenesis in vivo. (A-B) WT and *Nlrp3*^*-/-*^ BMDMs were infected with RVFV (MOI = 1) for 12h. (A) Immunoblot analysis of NLRP3, cleaved GSDMD in cell lysates. (B) IL-1β level in the supernatant were determined by ELISA. (C) Survival analysis of age-and sex- matched WT (n = 6) and *Nlrp3*^*-/-*^ (n = 6) mice infected intraperitoneally with 5 PFU of RVFV. (D-J) WT and *Nlrp3*^*-/-*^ mice were intraperitoneally infected with 5 PFU of RVFV and the serum, liver, and spleen samples were harvested at 2 days post-infection. (D) Viral loads in the serum were quantified by qRT-PCR. (E-F) Viral loads in livers (E) or spleens (F) were measured by plaque assay. (G) Serum level of IL-1β were measured by ELISA. (H) H&E staining of liver samples. Scale bar, 50 μm. The enlarged images indicate coalescing hepatocellular necrosis (a) and hepatic haemorrhage (b). (I-J) Immunohistochemistry staining of NP and IL-1β in livers (I) or spleens (J). Arrows indicate the infected-cells express IL-1β. Scale bar, 200 μm. Data are shown as mean ± SD from three independent experiments in (B). Statistical significance analysis was analyzed by two-way ANOVA in (B, G) or log-rank test in (C) or Student’s t-test in (D-F). *p < 0.05;**p < 0.01; ***p < 0.001; ****p < 0.0001; ns, no significance. Immunoblot results are representative of three independent experiments.

Finally, we investigated whether NLRP3 inflammasome activation contributed to RVFV pathogenesis in vivo. For this purpose, we performed RVFV infection in *Nlrp3*^*-/-*^ mice model compared to wild-type mice. We first analyzed whether NLRP3 deficiency affected RVFV triggered pyroptosis and IL-1β release in BMDMs harvested from wild-type (WT) and *Nlrp3*^*-/-*^ mice. Similar with the NLRP3 depleted THP-1^PMA^ cells, GSDMD cleavage and IL-1β release were reduced in RVFV-infected *Nlrp3*^*-/-*^ BMDMs compared to the infected WT BMDMs (Fig 7A and 7B). Furthermore, GSDMD cleavage and IL-1β release were also reduced in RVFV^RM^-infected BMDMs compared to RVFV^WT^-infected BMDMs, indicating that NSs mediated RVFV infection-triggered NLRP3-depdendent pyroptosis in these primary cells (S6A and S6B Fig). Next, we infected *Nlrp3*^*-/-*^ mice and sex/age-matched WT mice with 5 PFU of RVFV through intraperitoneal injection and monitored survival rate overtime. RVFV infection of *Nlrp3*^*-/-*^ mice resulted in a significantly lower mortality rate than infection of WT mice (Fig 7C). Since RVFV infection of WT mice led to rapid death since 3 days post infection (3 dpi), we performed RVFV infection and harvested tissue samples at an earlier time point (2 dpi) for analysis. Notably, the viral loads in the serum, liver, and spleen samples were comparable between RVFV infected *Nlrp3*^*-/-*^ mice and WT control mice (Fig 7D–7F). Meanwhile, the serum IL-1β level was lower in the RVFV-infected *Nlrp3*^*-/-*^ mice compared to the WT mice (Fig 7G). This indicated that the higher fatality rate observed in the infected WT mice may be associated with stronger inflammatory responses. Hematoxylin and eosin (HE) staining of the liver sections showed that more coagulative hepatocellular necrosis with inflammatory cells infiltration and hepatic hemorrhage were observed in the liver of RVFV-infected wild-type mice than in the infected *Nlrp3*^*-/-*^ mice (Fig 7H). Furthermore, staining with antibody against IL-1β and viral protein NP showed that RVFV infection led to significant IL-1β accumulation in both liver and spleen tissues, which were reduced in the infected *Nlrp3*^*-/-*^ mice (Fig 7I and 7J). To further verify the role of NLRP3 activation in RVFV pathogenesis, mice were infected with RVFV-NSs^RM^, which did not trigger NLRP3 activation. Similar with RVFV infection of the *Nlrp3*^*-/-*^ mouse model, RVFV-NSs^RM^ infection resulted in reduced fatality rate compared with the WT virus infection and the serum, liver, spleen viral loads were comparable between these two groups (S6C–S6F Fig). HE staining of the liver sections showed that RVFV-NSs^RM^-infected mice exhibited less coagulative hepatocellular necrosis compared with liver sections from mice infected by WT RVFV (S6G Fig). Staining with antibody against IL-1β and NP revealed that infection with RVFV-NSs^RM^ resulted in reduced IL-1β accumulation in both liver and spleen tissues compared with WT virus infection (S6H and S6I Fig). Taken together, these results supported that NLRP3 activation promotes RVFV triggered inflammatory pathogenesis in vivo.

## Discussion

While the mitochondria have been recognized as an important driver for inflammatory responses, whether the viruses regulate mitochondria homeostasis to trigger pathogenic inflammation remains elusive. Here, we identified a novel mechanism that induces ox-mtDNA release to activate the NLRP3 inflammasome during virus infection. This is triggered by the transcription inhibition effect of NSs that down-regulates MCL-1 abundance to activate the mitochondrial BAK leading to MOMP formation followed by mtROS accumulation and ox-mtDNA release into the cytosol. The cytoplasmic ox-mtDNA then binds and activates the NLRP3 inflammasome leading to inflammatory responses in RVFV infected cells. RVFV infection of mouse models showed that NLRP3 inflammasome activation promoted viral pathogenesis in vivo.

The transcription inhibition effect of NSs has been reported to suppress host anti-viral immune responses to promote viral replication [39]. Activation of pathogenic NLRP3 pyroptosis identified in this study represents another virulence mechanism associated with the transcription inhibition effect of NSs. In RVFV infected *Nlrp3*^*-/-*^ mouse, the virus titre was comparable with that from infected WT mouse at an early time point (2 days post-infection), indicating that the activated NLRP3 pyroptosis did not control viral replication but mainly contributed to inflammatory pathogenesis. Thus the transcription inhibition activity of NSs promotes both viral replication and pathogenic inflammatory responses, which would explain its critical role in mediating RVFV pathogenesis *in vivo*. It has been reported that other viruses, such as human papillomavirus, poliovirus, and influenza virus, also induce host transcription inhibition during infection. It is possible that these viruses may also trigger NLRP3-dependent inflammatory responses through activation of the mitochondrial MCL-1-BAK axis. In the meantime, whether NLRP3 could affect RVFV viral replication in the mouse model during later time-point could be investigated in the future study, since later downstream inflammatory responses mediated through NLRP3 could potentially affect viral infection.

A previous study reported that infection of murine dendritic cells with attenuated strains rMP-12 or RVFV deleted of NSs can trigger IL-1β release into the supernatant in the NLRP3-dependent manner only with LPS priming [40]. However, the underlying mechanisms of RVFV-triggered NLRP3 activation and whether NLRP3 pyroptosis contributed to RVFV pathogenesis *in vivo* was not characterized. Now we found here that RVFV infection THP-1^PMA^, without the need of LPS priming, can trigger NLRP3 inflammasome and robust IL-1β release. The discrepancy may be attributed to the different cell types employed in the two studies. Here we used a macrophage-like cell type, which represents a physiologically relevant target cell-type that is infected by RVFV *in vivo*. The relevance of NLRP3 activation in RVFV triggered pathogenesis is further confirmed in the *Nlrp3*^*-/-*^ mouse model. The sequence and functionality similarities between human and mouse NLRP3 proteins have been reported before [41,42]. Therefore, the NLRP3-pyroptosis mediated pathogenesis mechanism observed in the RVFV infected mouse model could potentially be correlated with the viral pathogenesis in the RVFV infected patients. It should also be noted that NSs could trigger apoptosis during RVFV infection and recently co-localization between NSs and caspase-3 has been reported [43,44]. Whether other cell death pathways, such as apoptosis, could contribute to RVFV pathogenesis, potentially in combination with pyroptosis, worth further investigation.

It has been reported that infection of SFTSV, another vector-borne bunyavirus that causes hemorrhagic fever, also triggers BAK-dependent NLRP3 activation that is associated with severe disease development and fatal infection in patients [32]. Similar with RVFV, monocyte/macrophage represents a target cell type infected by SFTSV that contributes to viral pathogenesis in clinical patients [32,45–48]. Different from RVFV, SFTSV induces up-regulation and auto-activation of BAK to trigger MOMP formation leading to ox-mtDNA release and NLRP3 activation [32]. Although the activation mechanisms are different, both viruses trigger BAK-dependent mitochondrial dysfunction followed by NLRP3-pyroptosis inflammatory responses. Since a number of vector-borne viruses target monocyte/macrophage cells in the peripheral blood for infection, whether the BAK-dependent NLRP3 activation represents a common inflammatory pathogenesis mechanism triggered by these viruses could be investigated in the future.

## Materials and methods

### Ethics statement

All animal experiments were conducted following the Ministry of Health national guidelines for housing and care of laboratory animals and performed in accordance with institutional regulations after review and approved by Wuhan Institute of Virology animal welfare committee, Chinese Academy of Sciences (Wuhan, China). The approval number was WIVA38202207.

### Cell lines

HEK293T, Vero, BSRT/7 and BHK-21 were cultured in Dulbecco’s modified Eagle’s medium (DMEM; GIBCO) containing 10% fetal bovine serum (FBS; GIBCO),1% penicillin/streptomycin (GIBCO). THP-1 cells were cultured in RPMI-1640 medium containing 10% FBS (GIBCO), 1% penicillin/streptomycin (GIBCO). All cells were cultured at 37°C in a humidified incubator containing 5% CO_2_. HEK293T, Vero, THP-1 were obtained from American Type Culture Collection (ATCC).

### Bone marrow-derived macrophage (BMDM) generation

Primary mouse BMDMs from wild-type (WT) and *Nlrp3*^*-/*-^ mice were grown for 6 days in RPMI 1640 medium supplemented with 10% heat-inactivated fetal bovine serum, 1% penicillin/streptomycin, murine macrophage colony-stimulating factor (M-CSF; 20 ng/ml). BMDMs were seeded in 12-well plates at a density of 1 x 10^6^ cells/well.

### Viruses and infection

The recombinant wild type Rift Valley fever virus (RVFV) strain BJ01, NSs deletion virus (RVFV^ΔNSs^), NSs mutant virus (R16H and M250K, RVFV-NSs^RM^) were generated by reverse genetics as previously described [10]. Viral titer was determined by plaque assay as described previously [49]. For THP-1^PMA^ infection, THP-1 cells were differentiated into macrophages with 40 ng/mL PMA (Sigma) for 24 h, and cells were cultured without PMA for 24 h. The differentiated cells were then infected with virus. For BMDMs infection, BMDMs were first primed with LPS (500 ng/mL) for 4 h, then the supernatant was removed and infected with virus at an MOI of 1 for 12 h. All RVFV infection experiments were performed under biosafety level 3 (BSL-3) conditions.

### Antibody and Reagents

Anti-NLRP3 (#13158), anti-cleaved-caspase-1 (#4199), anti-GSDMD (#97558), anti-MLKL (#14993), anti-pMLKL (S358, #91689), anti-MCL-1(#94296), anti-BAK (used in western blot, #12105) antibodies were purchased from Cell Signaling Technology. Anti-NLRP3 (used in western blot analysis of cell lysates from BMDMs, #AG-20B-0014) was purchased from AdipoGen.Anti-IL-1β (#A16288), anti-BAK (used in immunofluorescence assay, #A0498), anti-BAX (#A12009), anti-BCL-xL (#A19703), anti-DNA Polymerase gamma (POLG, #A1323) were obtained from ABclonal (Wuhan, China). Polyclonal rabbit anti-streptavidin tag (A00626) was obtained from GenScript. Anti-GSDMD (#AB209845) was obtained from Abcam.Anti-ASC (#sc-271054), anti-8-OH-dG (#sc-66036) were purchased from Santa Cruz Biotechnology. Anti-caspase-1(#22915-1-AP), anti-Alpha Tubulin (#11224-1-AP) were purchased from Proteintech. Rabbit anti-RVFV-NP, anti-RVFV-NSs were made in-house.

VX-765 (S222), MCC950 (S7809), Z-VAD-FMK (S7023) were obtained from Selleck. SM-164 (HY-15989), staurosporine (HY-15141) was obtained from MedChemExpress. LPS (L4391), CCCP (C2759), PMA (P1585), DSS (S1885), Digitonin (D141) were purchased from Sigma-Aldrich. MitoSOX (M36008), TMRM (T668) were purchased from Thermo Fisher. PI (Propidium Iodide, ST511), and DAPI (4,6-diamidino-2-phenylindole, C1002) were purchased from Beyotime. Murine M-CSF (315–02) was purchased from PeproTech.

### Plasmids and transient expression

RVFV NP, NSs, RdRp, Gn and Gc genes were PCR-amplified from viral cDNAs with specific primers, and cloned into the pRK plasmid vector with a C-terminal or N-terminal strep tag. All plasmids were verified by sequencing. HEK293T cells were transfected with 1 μg viral protein-expressing plasmid using Lipofectamine 2000 (Thermo Fisher Scientific, 11668019), and the cell lysates were collected at 24 h post transfection.

### Generation of knockdown and knockout cell lines

A PLKO.1 vector encoding shRNAs or lentiCRISPRv2 vector encoding gRNAs was transfected into HEK293T cells with pCMV-dR8.91 and pMD2.G plasmids at a 2/3/1 ratio to generate knockdown, knockout lentiviral particles, respectively. Lentiviral particles were collected 48 h later and centrifuged at 3000 g for 5 min, filtered through a 0.45-μm filter. Cells were transduced with lentiviral particles for 24 h and then selected with 2 μg/ml puromycin. Sequences of targeting shRNA or sgRNA used in this study are shown in Supporting information, S1 and S2 Tables respectively.

### Generation of cell lines stably expressing MCL-1

Full-length human MCL-1 was PCR-amplified from THP-1 cells cDNAs and inserted into the pcDH-CMV lentiviral vector. A Flag-tag was added to the N terminus of human MCL-1. Lentiviral particles were produced and THP-1 cells were transduced as described above. All plasmids were verified by sequencing.

### RNA isolation and quantitative RT-PCR

For cell samples, total RNA were extracted with TRIzol Reagent (Thermo Fisher Scientific, 15596026). For mice serum samples, total RNA were extracted with Viral RNA/DNA Extraction Kit (Takara, Japan) according to the manufacturer’s instructions. cDNA was synthesized by using the HiScriptIII 1^st^ Strand cDNA Synthesis Kit (Vazyme, China). Quantitative RT-PCR was performed with ChamQ Universal SYBR quantitative PCR Master Mix (Vazyme, China). Data were normalized to 18S rRNA expression, and relative expression changes were analyzed according to the 2^-ΔΔCT^ method. Primer sequences are shown in Supporting information, S3 Table.

### Western blotting

Cells were collected with lysis buffer (P0013, Beyotime). We usually loaded whole cell lysates around 20μg per lane to detect the expression of pMLKL, full GSDMD, and cleaved GSDMD. For detecting other proteins, we loaded whole cell lysates around 10μg per lane for western blot analysis. Cell lysates were subjected to 10–15% SDS–PAGE gel and transferred to 0.2 μm PVDF membranes (Millipore). The membrane was blocked with 5% non-fat milk in TBST for 1 h at room temperature and incubated with indicated primary antibodies overnight at 4°C. The membrane was washed three times with TBST and then incubated with HRP-conjugated goat secondary antibodies (Proteintech) for 1h at room temperature. Protein levels were detected with enhanced chemiluminescence (ECL) kit (Millipore) and imaged using a Chemiluminescence Analyzer (Chemiscope600pro).

### ELISA

IL-1β level was analyzed using IL-1β ELISA Kit (RD systems) or mouse IL-1β ELISA Kit (BioLgend) according to the manufacturer’s instructions.

### Mature IL-1β detection

The mature IL-1β (P17) in cell culture supernatants were detected as previously described [50]. Briefly, an equal volume of methanol and 0.25 volumes of chloroform were added to the supernatants to precipitate the proteins. The supernatant mixtures were vortexed and then centrifuged at 16000 g for 15 min at 4°C. The upper phase was removed, and then 500 μl methanol was added to the interphase. The mixtures were vortexed and centrifuged at 16000 g for 15min, and the protein pellets were dried at 55°C. The protein pellets were mixed with SDS loading buffer for western blotting analysis.

### ASC oligomerization detection

ASC oligomers were detected as previously described [32]. Briefly, infected THP-1^PMA^ cells were lysed by 1% NP40 lysis buffer, and cell lysates were centrifugated at 6000 rpm for 15 min at 4°C. The pellets were washed with PBS for 3 times and cross-linked with 4 mM Disuccinimidyl suberate (DSS) at 37°C for 30 min. The cross-linked pellets were centrifuged and mixed with SDS loading buffer for western blotting analysis.

### Mitochondrial DNA isolation and quantification

The total and cytosolic DNA extraction was performed as described previously by using FastPure Cell/Tissue DNA Isolation Mini Kit (Vazyme, China) [32]. Quantitative PCR was performed to quantify mtDNA level from extracted DNA with specific primers. The levels of total mtDNA were calculated as mtDNA normalized to nGAPDH. For cytosolic mtDNA detection, 20 ng of a purified plasmid encoding EGFP gene was added to the eluted solution as described previously [51]. The relative content of cytosolic mtDNA was normalized to EGFP. The relative content of cytosolic versus total mtDNA was calculated. Primer sequences are shown in Supporting information, S3 Table.

### Immunoprecipitation

THP-1^PMA^ cells were infected with RVFV (MOI = 5) for 12 h, and collected in lysis buffer (P0013D, Beyotime) supplemented with protease inhibitor cocktail (Roche) for 20 min at 4°C. Cell lysates were centrifuged at 10000 g for 10 min at 4°C. The supernatant was collected and incubated with anti-NLRP3 antibody at 4°C overnight. Magnetic beads (Thermo Fisher Scientific, 88804) were then added and incubated at room temperature for 1 h. After three times wash, fractions bound to beads were eluted. DNA precipitation was extracted and the mtDNA level was analyzed as described above.

### Cell death assay

For cell death assay, cell culture supernatants were harvested to access the cell death by using the LDH Cytotoxicity Assay Kit (C0017, Beyotime) according to the manufacturer’s instructions. For PI staining, cells were incubated with 1 μg/ml Propidium iodide (Beyotime) for 30 min at 37°C and PI fluorescence was measured by an epifluorescence microscope (Olympus IX73).

### Measurement of mitochondrial membrane potential and ROS

Cells were incubated with 100 nM TMRM (Invitrogen) in culture medium at 37°C for 30 min or 5 μM MitoSOX (Invirtogen) for 20 min and then washed one time with PBS. TMRM fluorescence was measured with a multimode microplate reader (Perkin Elmer). MitoSOX fluorescence was measured by an epifluorescence microscope (Olympus IX73).

### Immunofluorescence assay (IFA)

Infected cells were washed with PBS and fixed with 4% PFA at room temperature for 30 min, then permeabilized with 0.2% Triton X-100 for 10 min, and the non-specific binding sites were blocked with 3% bovine serum albumin (BSA) for 30min. Incubation with indicated primary antibodies for 2 h at room temperature, followed by extensive washing and incubation with secondary antibodies for 1 h at room temperature. Subsequently, cells were stained with DAPI (Beyotime, China) for 10 min. Image was taken and analyzed by confocal microscope (Andor Dragonfly 202).

### Animal study

The C57BL/6J wild-type mice were purchased from Beijing Vital River Laboratory Animal Technology. The *Nlrp3*^*−/−*^ mice were kindly provide by Prof. Chaohong Liu, Huazhong University of Science and Technology. For *Nlrp3*^*-/-*^ mice infection, age- and sex-matched, 8- to 12-week-old wild-type and *Nlrp3*^*-/-*^ mice were intraperitoneally injected with 5 PFU of RVFV in 100 μL PBS. For RVFV^RM^ infection, age- and sex-matched, 8-week-old wild-type mice were intraperitoneally injected with 5 PFU of RVFV or RVFV^RM^. Mice were monitored daily for clinical symptoms and weighted. Serum, liver, and spleen samples were collected at the indicated time for analysis.

### Histopathology and immunohistochemistry

Liver and spleen samples collected at 2 dpi were fixed in 4% paraformaldehyde, embedded in paraffin wax, and cut into sections of 4 μm. The sections were further used for routine histology stained with hematoxylin and eosin (H&E) or immunohistochemistry (IHC) using antibodies against mouse IL-1β and viral protein NP.

### Statistical analysis

All statistical analyses were performed using GraphPad Prism 9 software. The data are shown as mean ± SD. Statistical significance was determined by two-tailed Student’s t-test, one-way ANOVA or two-way ANOVA as indicated in figure legends. The log-rank test was used to compare the survival curves. A p value < 0.05 was considered statistically significant. *p < 0.05; **p < 0.01; ***p < 0.001; ****p < 0.0001; ns, no significance.

## Supporting information

S1 Fig RVFV infection induces cell death. **Related to Fig 1.** (A and B) Representative images of cell death determined by PI staining in THP-1^PMA^ cells infected with RVFV (MOI = 5) with indicated time (A) and indicated MOI for 24 h (B). BF, bright field. Scale bar,100 μm. (C and D) Immunoblot analysis of pMLKL in THP-1^PMA^ cells treated with L/S/Z (1 μg/mL LPS, 2.5 μM SM-164, 100 μM Z-VAD) for 6 h, or infected with RVFV (MOI = 5) with indicated time (C) and indicated MOI for 24 h (D). Immunoblot results are representative of three independent experiments.
(TIF)

S2 Fig RVFV infection activates NLRP3-GSDMD dependent pyroptosis. **Related to Fig 2.** (A-C) THP-1^PMA^ cells stably expressing non-targeting shRNA (sh-Ctrl) or shRNAs against GSDMD were infected with RVFV (MOI = 5) for 24 h. (A) Immunoblot analysis of P17 level in supernatants and GSDMD expression in cell lysates. (B) IL-1β release was quantified by ELISA. (C) Cell death was determined by LDH release. Data are shown as mean ± SD from three independent experiments. Statistical significance was analyzed by two-way ANOVA. ****p < 0.0001. Immunoblot results are representative of three independent experiments.
(TIF)

S3 Fig BAX is dispensible for RVFV-induced pyroptosis. **Related to Fig 4.** (A) THP-1^PMA^ cells were treated with Staurosporine (STP, 1μM, 6 h) or infected with RVFV (MOI = 10) for 12 h. Cells were co-stained for BAK (green), Tomm20 (red), and DAPI (blue) for immunofluorescence. Arrow indicates aggregated BAK signals. Scale bar,10 μm. (B) THP-1 cells stably expressing non-targeting sgRNA (sg-Ctrl) or sgRNAs against BAX infected with RVFV (MOI = 5) for 12h. Cytosolic mtDNA levels were determined with qPCR. (C-E) THP-1 cells stably expressing non-targeting sgRNA (sg-Ctrl) or sgRNAs against BAX infected with RVFV (MOI = 5) for 24 h. (C) LDH release was quantified. (D) IL-1β release was quantified by ELISA. (E) Immunoblot analysis of cleaved GSDMD, BAX, NP expression in cell lysates. Data are shown as mean ± SD from three independent experiments. Statistical significance was analyzed by two-way ANOVA. ns, no significance. Immunoblot results are representative of three independent experiments.
(TIF)

S4 Fig RVFV NSs induces down-regulation of MCL-1 and cell death. **Related to Fig 5.** (A-B) THP-1^PMA^ cells were infected with RVFV^WT^ or RVFV^△NSs^ (MOI = 5) for 6,12 h. (A) Immunoblot analysis of MCL-1, NSs, NP expression in the cell lysates. (B) Cell death was determined by LDH release. Data are shown as mean ± SD from three independent experiments. Statistical significance was analyzed by Student’s t-test. **p < 0.01; ***p<0.001; ****p < 0.0001. Immunoblot results are representative of three independent experiments.
(TIF)

S5 Fig RVFV NSs down-regulates MCL-1 and promotes mtROS production. **Related to Fig 6.** (A) THP-1^PMA^ cells were infected with RVFV^WT^ or RVFV^△NSs^ (MOI = 5) for 6,12 h and intracellular mRNA level of MCL-1 was measured by qRT-PCR. (B) THP-1^PMA^ cells were infected with RVFV^WT^ or RVFV-NSs^RM^ (RVFV^RM^, MOI = 5) for 12 h. Intracellular mtROS was stained with MitoSOX. Scale bar, 100 μm. Data are shown as mean ± SD from three independent experiments. Statistical significance was analyzed by Student’s t-test. **p < 0.01; ****p < 0.0001.
(TIF)

S6 Fig RVFV-NSs^RM^ virus showed alleviated viral pathogenesis in vivo. **Related to Fig 7.** (A-B) BMDMs were infected with RVFV^WT^ or RVFV^RM^ (MOI = 1) for 12h. (A) Immunoblot analysis of cleaved GSDMD, NP in cell lysates. (B)IL-1β release into the supernatant was quantified by ELISA. (C) Survival analysis of age- and sex- matched WT mice (n = 10/group) infected intraperitoneally with 5 PFU of RVFV^WT^ or RVFV^RM^. (D-F) WT mice were intraperitoneally infected with 5 PFU of RVFV^WT^ or RVFV-NSs^RM^ (RVFV^RM^) and the serum, liver, spleen samples were harvested at 2 days post infection. (D) Viral loads in the serum were quantified by qRT-PCR.(E-F) Viral titers were measured in livers (E) and spleens (F) by plaque assay. (G) H&E staining of liver samples. Scale bar, 50 μm. The enlarged images indicate coalescing hepatocellular necrosis. (H-I) Immunohistochemistry staining of NP and IL-1β in livers (H) and spleens (I). Arrows indicate the infected-cells express IL-1β. Scale bar, 200 μm. Data are shown as mean ± SD from three independent experiments in (B). Statistical significance analysis was analyzed by Student’s t-test in (B, D-F) or log-rank test in (C). *p < 0.05; ns, no significance. Immunoblot results are representative of three independent experiments.
(TIF)

S7 Fig A proposed model of RVFV triggered NLRP3 inflammasome activation. Created with BioRender.com.
(TIF)

S1 Data Source data used to generate graphs in Fig 3C. (XLSX)

S1 Table shRNA sequences for knockdown. (DOCX)

S2 Table sgRNA sequences for knockout. (DOCX)

S3 Table Primers for qRT-PCR. (DOCX)
