# Correction: RVFV virulence factor NSs triggers the mitochondrial MCL-1-BAK axis to activate pathogenic NLRP3 pyroptosis

**DOI:** 10.1371/journal.ppat.1013113

**Published:** 2025-04-24

**Authors:** Zhenqiong Guan, Huiling Li, Chongtao Zhang, Ziyan Huang, Meidi Ye, Yulan Zhang, Shufen Li, Ke Peng

S1 Fig is incorrect. Please view the correct S1 Fig below.

## Supporting information

S1 Fig RVFV infection induces cell death. Related to Fig 1. (A and B) Representative images of cell death determined by PI staining in THP-1^PMA^ cells infected with RVFV (MOI = 5) with indicated time (A) and indicated MOI for 24 h (B). BF, bright field. Scale bar, 100 μm. (C and D) Immunoblot analysis of pMLKL in THP-1^PMA^ cells treated with L/S/Z (1 μg/mL LPS, 2.5 μM SM-164, 100 μM Z-VAD) for 6 h, or infected with RVFV (MOI = 5) with indicated time (C) and indicated MOI for 24 h (D). Immunoblot results are representative of three independent experiments.
(JPEG)
