# Supplementary figures and images for: RVFV virulence factor NSs triggers the mitochondrial MCL-1-BAK axis to activate pathogenic NLRP3 pyroptosis
Source: PLoS Pathog. 2024 Aug 30;20(8):e1012387. doi: 10.1371/journal.ppat.1012387 (PMC11364418; doi:10.1371/journal.ppat.1012387)

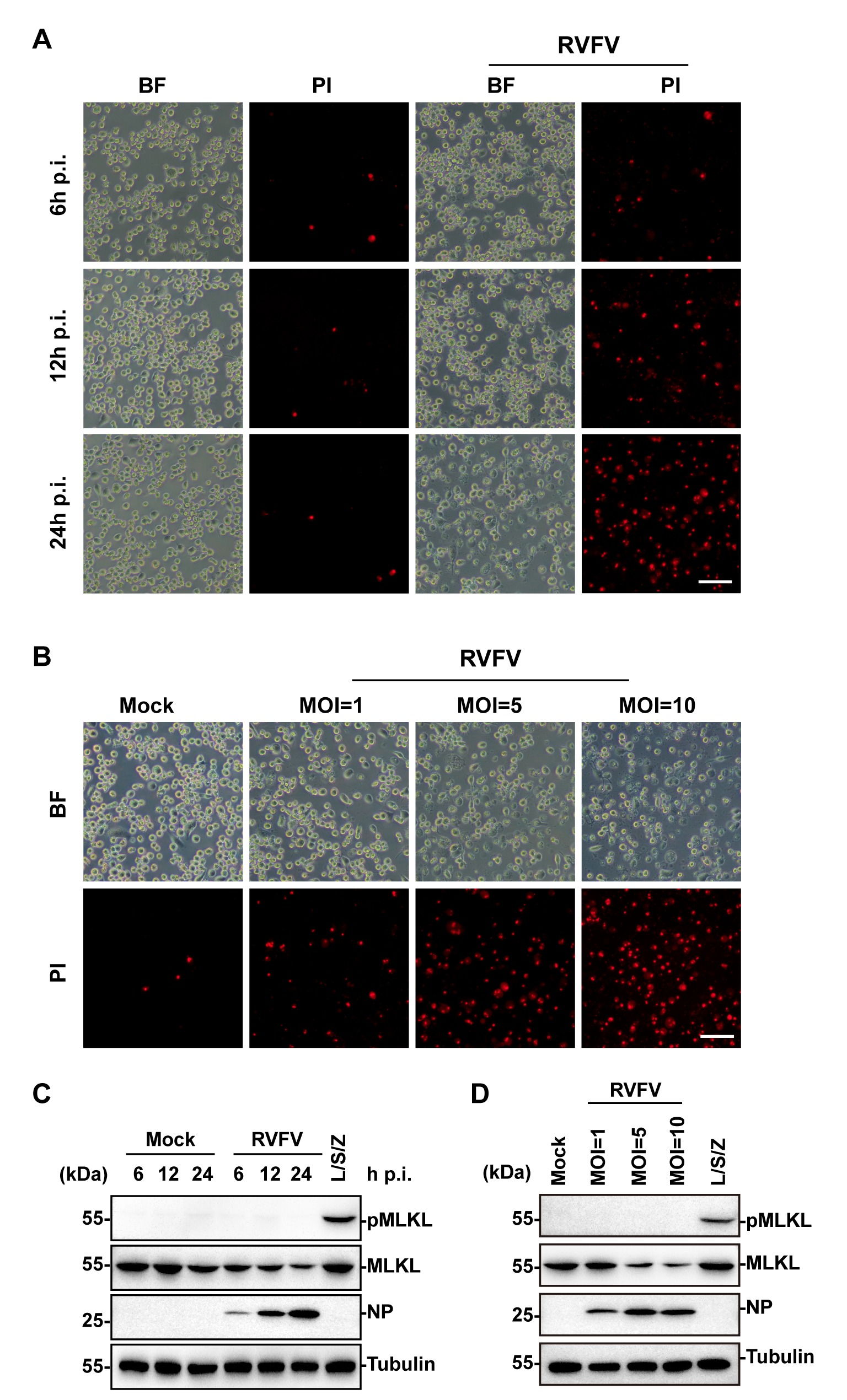

Supplement: S1 Fig — Related to Fig 1. (A and B) Representative images of cell death determined by PI staining in THP-1PMA cells infected with RVFV (MOI = 5) with indicated time (A) and indicated MOI for 24 h (B). BF, bright field. Scale bar,100 μm. (C and D) Immunoblot analysis of pMLKL in THP-1PMA cells treated with L/S/Z (1 μg/mL LPS, 2.5 μM SM-164, 100 μM Z-VAD) for 6 h, or infected with RVFV (MOI = 5) with indicated time (C) and indicated MOI for 24 h (D). Immunoblot results are representative of three independent experiments. (TIF) [file ppat.1012387.s001.tif]

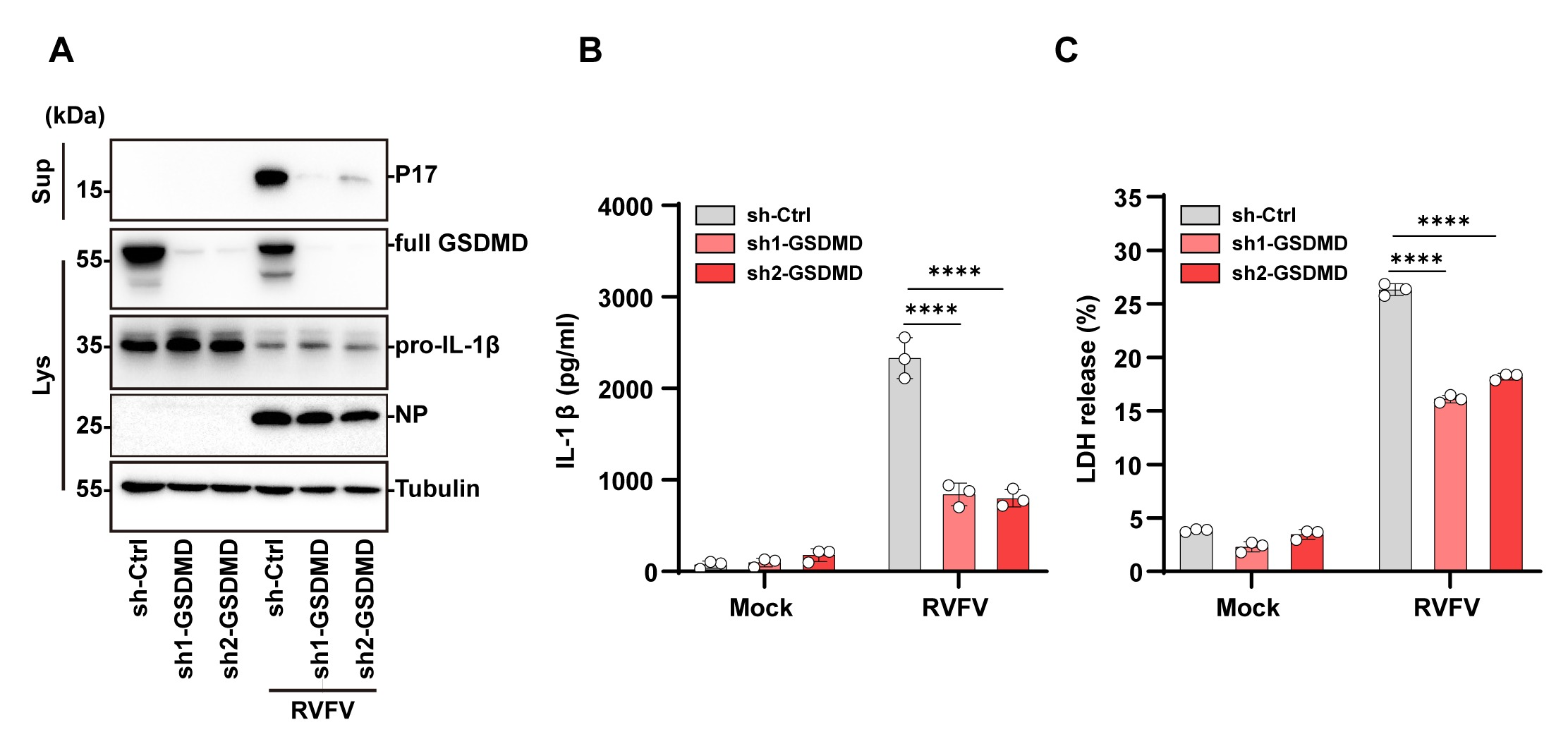

Supplement: S2 Fig — Related to Fig 2. (A-C) THP-1PMA cells stably expressing non-targeting shRNA (sh-Ctrl) or shRNAs against GSDMD were infected with RVFV (MOI = 5) for 24 h. (A) Immunoblot analysis of P17 level in supernatants and GSDMD expression in cell lysates. (B) IL-1β release was quantified by ELISA. (C) Cell death was determined by LDH release. Data are shown as mean ± SD from three independent experiments. Statistical significance was analyzed by two-way ANOVA. ****p < 0.0001. Immunoblot results are representative of three independent experiments. (TIF) [file ppat.1012387.s002.tif]

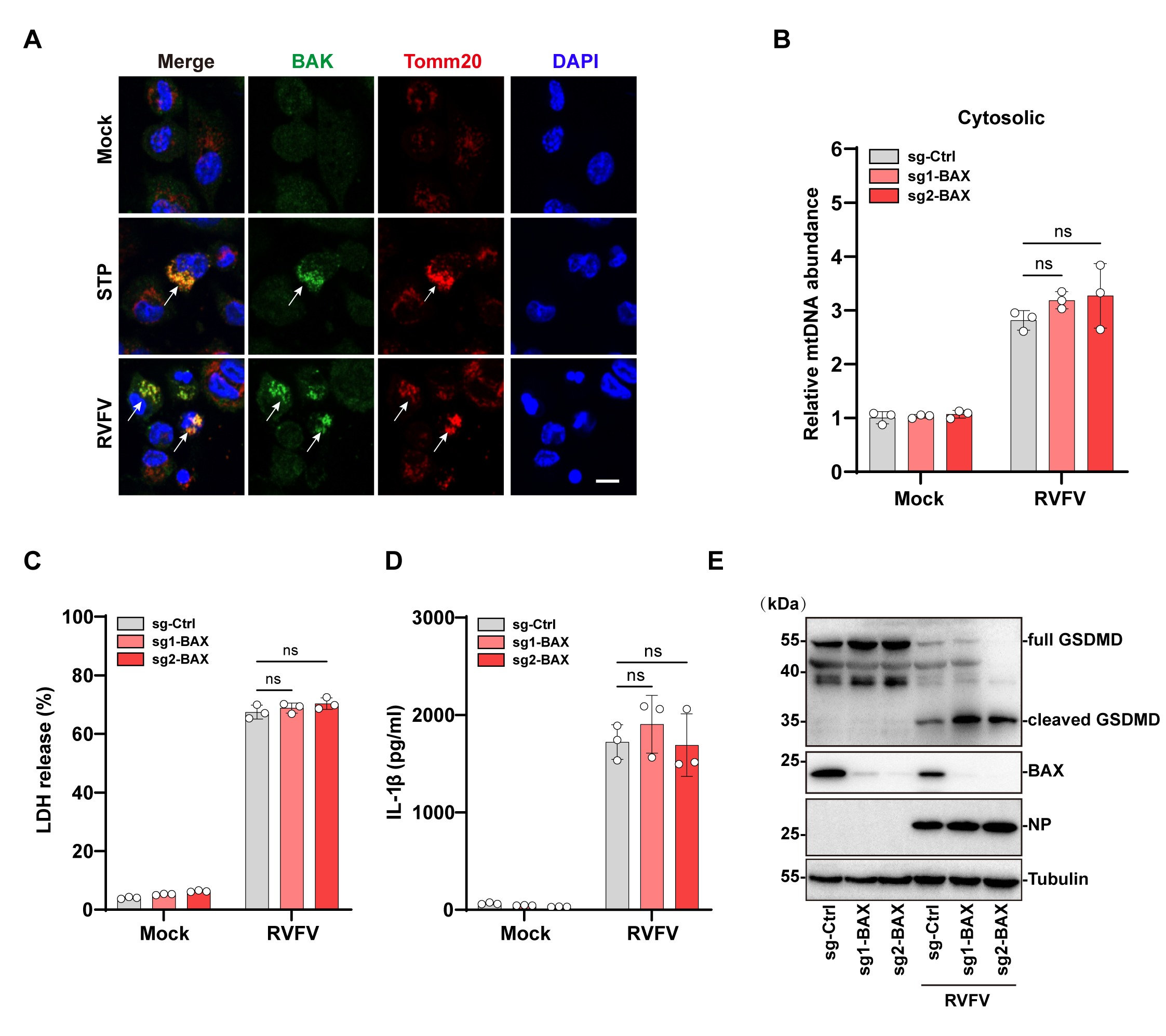

Supplement: S3 Fig — Related to Fig 4. (A) THP-1PMA cells were treated with Staurosporine (STP, 1μM, 6 h) or infected with RVFV (MOI = 10) for 12 h. Cells were co-stained for BAK (green), Tomm20 (red), and DAPI (blue) for immunofluorescence. Arrow indicates aggregated BAK signals. Scale bar,10 μm. (B) THP-1 cells stably expressing non-targeting sgRNA (sg-Ctrl) or sgRNAs against BAX infected with RVFV (MOI = 5) for 12h. Cytosolic mtDNA levels were determined with qPCR. (C-E) THP-1 cells stably expressing non-targeting sgRNA (sg-Ctrl) or sgRNAs against BAX infected with RVFV (MOI = 5) for 24 h. (C) LDH release was quantified. (D) IL-1β release was quantified by ELISA. (E) Immunoblot analysis of cleaved GSDMD, BAX, NP expression in cell lysates. Data are shown as mean ± SD from three independent experiments. Statistical significance was analyzed by two-way ANOVA. ns, no significance. Immunoblot results are representative of three independent experiments. (TIF) [file ppat.1012387.s003.tif]

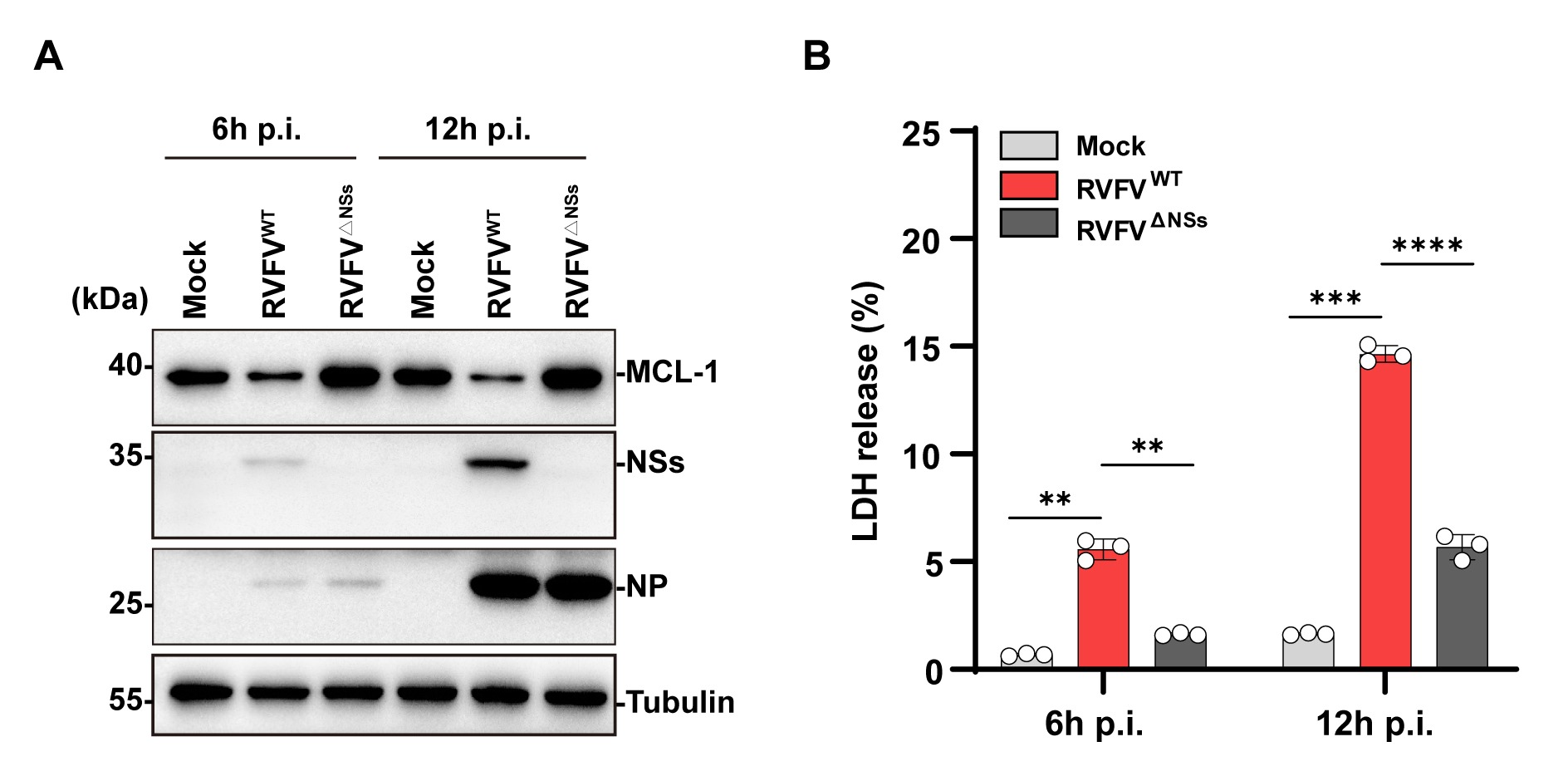

Supplement: S4 Fig — Related to Fig 5. (A-B) THP-1PMA cells were infected with RVFVWT or RVFV△NSs (MOI = 5) for 6,12 h. (A) Immunoblot analysis of MCL-1, NSs, NP expression in the cell lysates. (B) Cell death was determined by LDH release. Data are shown as mean ± SD from three independent experiments. Statistical significance was analyzed by Student’s t-test. **p < 0.01; ***p<0.001; ****p < 0.0001. Immunoblot results are representative of three independent experiments. (TIF) [file ppat.1012387.s004.tif]

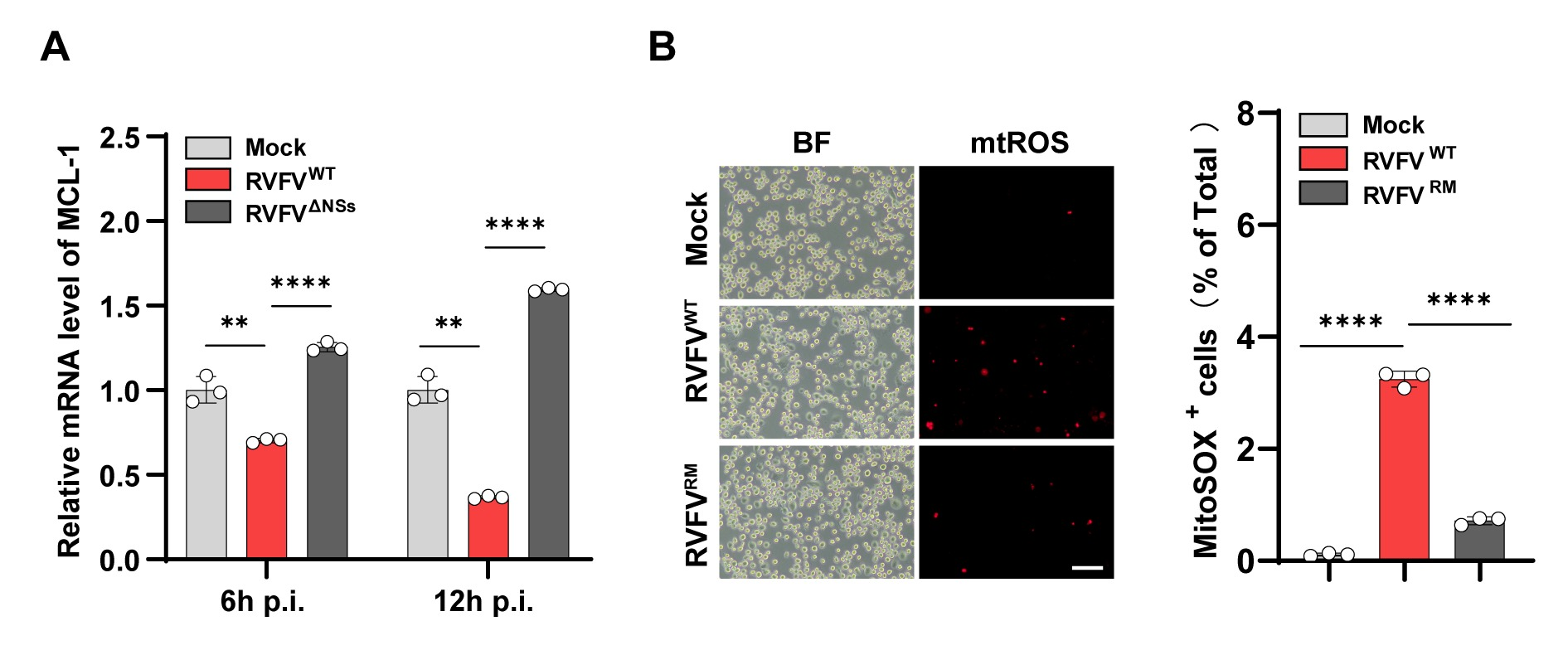

Supplement: S5 Fig — Related to Fig 6. (A) THP-1PMA cells were infected with RVFVWT or RVFV△NSs (MOI = 5) for 6,12 h and intracellular mRNA level of MCL-1 was measured by qRT-PCR. (B) THP-1PMA cells were infected with RVFVWT or RVFV-NSsRM (RVFVRM, MOI = 5) for 12 h. Intracellular mtROS was stained with MitoSOX. Scale bar, 100 μm. Data are shown as mean ± SD from three independent experiments. Statistical significance was analyzed by Student’s t-test. **p < 0.01; ****p < 0.0001. (TIF) [file ppat.1012387.s005.tif]

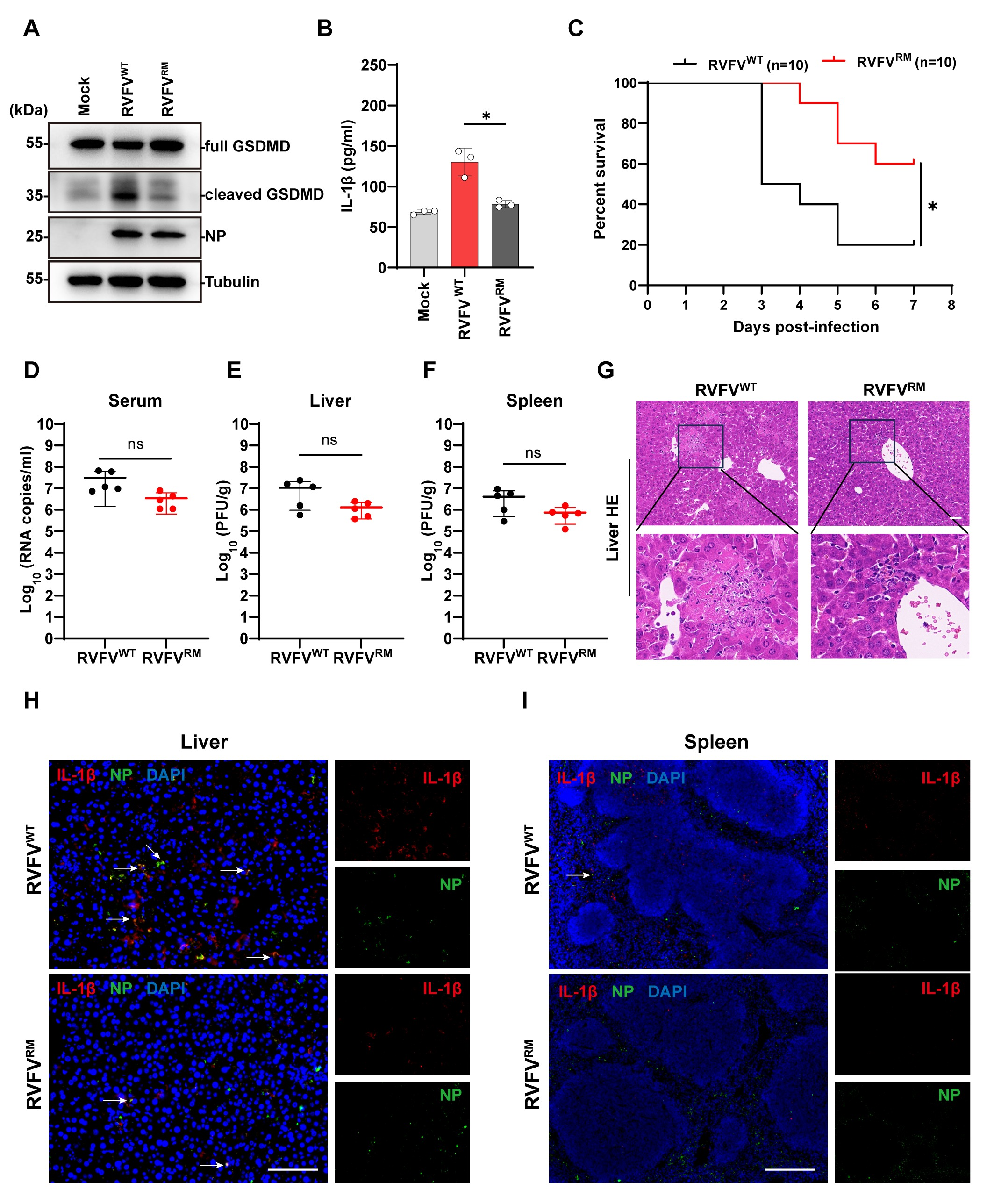

Supplement: S6 Fig — Related to Fig 7. (A-B) BMDMs were infected with RVFVWT or RVFVRM (MOI = 1) for 12h. (A) Immunoblot analysis of cleaved GSDMD, NP in cell lysates. (B)IL-1β release into the supernatant was quantified by ELISA. (C) Survival analysis of age- and sex- matched WT mice (n = 10/group) infected intraperitoneally with 5 PFU of RVFVWT or RVFVRM. (D-F) WT mice were intraperitoneally infected with 5 PFU of RVFVWT or RVFV-NSsRM (RVFVRM) and the serum, liver, spleen samples were harvested at 2 days post infection. (D) Viral loads in the serum were quantified by qRT-PCR.(E-F) Viral titers were measured in livers (E) and spleens (F) by plaque assay. (G) H&E staining of liver samples. Scale bar, 50 μm. The enlarged images indicate coalescing hepatocellular necrosis. (H-I) Immunohistochemistry staining of NP and IL-1β in livers (H) and spleens (I). Arrows indicate the infected-cells express IL-1β. Scale bar, 200 μm. Data are shown as mean ± SD from three independent experiments in (B). Statistical significance analysis was analyzed by Student’s t-test in (B, D-F) or log-rank test in (C). *p < 0.05; ns, no significance. Immunoblot results are representative of three independent experiments. (TIF) [file ppat.1012387.s006.tif]

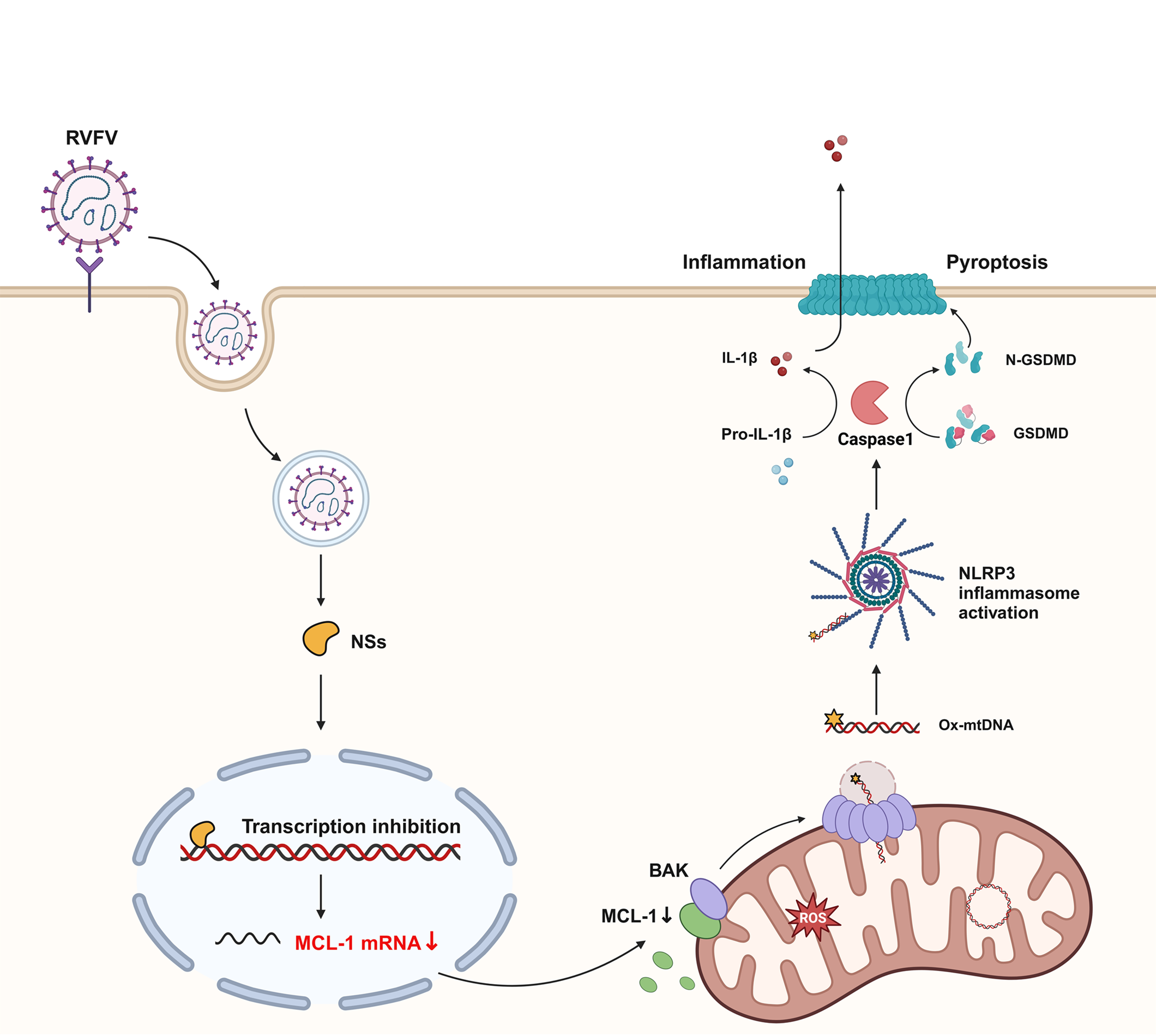

Supplement: S7 Fig — Created with BioRender.com. (TIF) [file ppat.1012387.s007.tif]
