# Supplementary material for: RVFV virulence factor NSs triggers the mitochondrial MCL-1-BAK axis to activate pathogenic NLRP3 pyroptosis
Source: PLoS Pathog. 2024 Aug 30;20(8):e1012387. doi: 10.1371/journal.ppat.1012387 (PMC11364418; doi:10.1371/journal.ppat.1012387)
Supplement: S1 Table — (DOCX) [file ppat.1012387.s009.docx]

**S1 Table. shRNA sequences for knockdown.**

| **Gene** | **Forward primer (5’-3’)** | **Reverse primer (5’-3’)** |
| --- | --- | --- |
| Human NLRP3 | GTGGATCTAGCCACGCTAATG | CATTAGCGTGGCTAGATCCAC |
| Human ASC | GCCCACCAACCCAAGCAAGAT | ATCTTGCTTGGGTTGGTGGGC |
| Human Caspase1 | CACACGTCTTGCTCTCATTAT | ATAATGAGAGCAAGACGTGTG |
| Human  GSDMD sh1 | GTGTGTCAACCTGTCTATCAA | TTGATAGACAGGTTGACACAC |
| Human  GSDMD sh2 | CAGCACCTCAATGAATGTGTA | TACACATTCATTGAGGTGCTG |
| Human  POLG sh1 | GCGCTTACTAATGCAGTTTAA | TTAAACTGCATTAGTAAGCGC |
| Human  POLG sh2 | TTCCTTTGACCGAGCTCATAT | ATATGAGCTCGGTCAAAGGAA |
