# Supplementary material for: RVFV virulence factor NSs triggers the mitochondrial MCL-1-BAK axis to activate pathogenic NLRP3 pyroptosis
Source: PLoS Pathog. 2024 Aug 30;20(8):e1012387. doi: 10.1371/journal.ppat.1012387 (PMC11364418; doi:10.1371/journal.ppat.1012387)
Supplement: S2 Table — (DOCX) [file ppat.1012387.s010.docx]

**S2 Table. sgRNA sequences for knockout.**

| **Gene** | **Forward primer (5’-3’)** | **Reverse primer (5’-3’)** |
| --- | --- | --- |
| Human BAK sg1 | TGCATCACTGCATTGCCCGG | CCGGGCAATGCAGTGATGCA |
| Human BAK sg2 | AGACCTGAAAAATGGCTTCG | CGAAGCCATTTTTCAGGTCT |
| Human BAX sg1 | TCGGAAAAAGACCTCTCGGG | CCCGAGAGGTCTTTTTCCGA |
| Human BAX sg2 | TTGGGCTGGATCCAAGACCA | TGGTCTTGGATCCAGCCCAA |
