# Supplementary material for: RVFV virulence factor NSs triggers the mitochondrial MCL-1-BAK axis to activate pathogenic NLRP3 pyroptosis
Source: PLoS Pathog. 2024 Aug 30;20(8):e1012387. doi: 10.1371/journal.ppat.1012387 (PMC11364418; doi:10.1371/journal.ppat.1012387)
Supplement: S3 Table — (DOCX) [file ppat.1012387.s011.docx]

**S3 Table. Primers for qRT-PCR used in this study.**

| **Gene** | **Forward primer (5’-3’)** | **Reverse primer (5’-3’)** |
| --- | --- | --- |
| Human  18S rRNA | TGAGAAACGGCTACCACATC | TTACAGGGCCTCGAAAGAGT |
| Human  MCL-1 | TGCTTCGGAAACTGGACATCA | TAGCCACAAAGGCACCAAAAG |
| Human  nGAPDH | CAATGACCCCTTCATTGACC | TGGAAGATGGTGATGGGATT |
| Human  mtDNA | CACCCAAGAACAGGGTTTGT | TGGCCATGGGTATGTTGTTAA |
| EGFP | ACGGCGACGTAAACGGCCAC | GTCAGGGTGGTCACGAGGGT |
| RVFV NP | TAAGGGCGATATTGGATGCT | TTGCAGCAACTTCCTCCTTT |
