# Supplementary figures and images for: Correction: RVFV virulence factor NSs triggers the mitochondrial MCL-1-BAK axis to activate pathogenic NLRP3 pyroptosis
Source: PLoS Pathog. 2025 Apr 24;21(4):e1013113. doi: 10.1371/journal.ppat.1013113 (PMC12021176; doi:10.1371/journal.ppat.1013113)

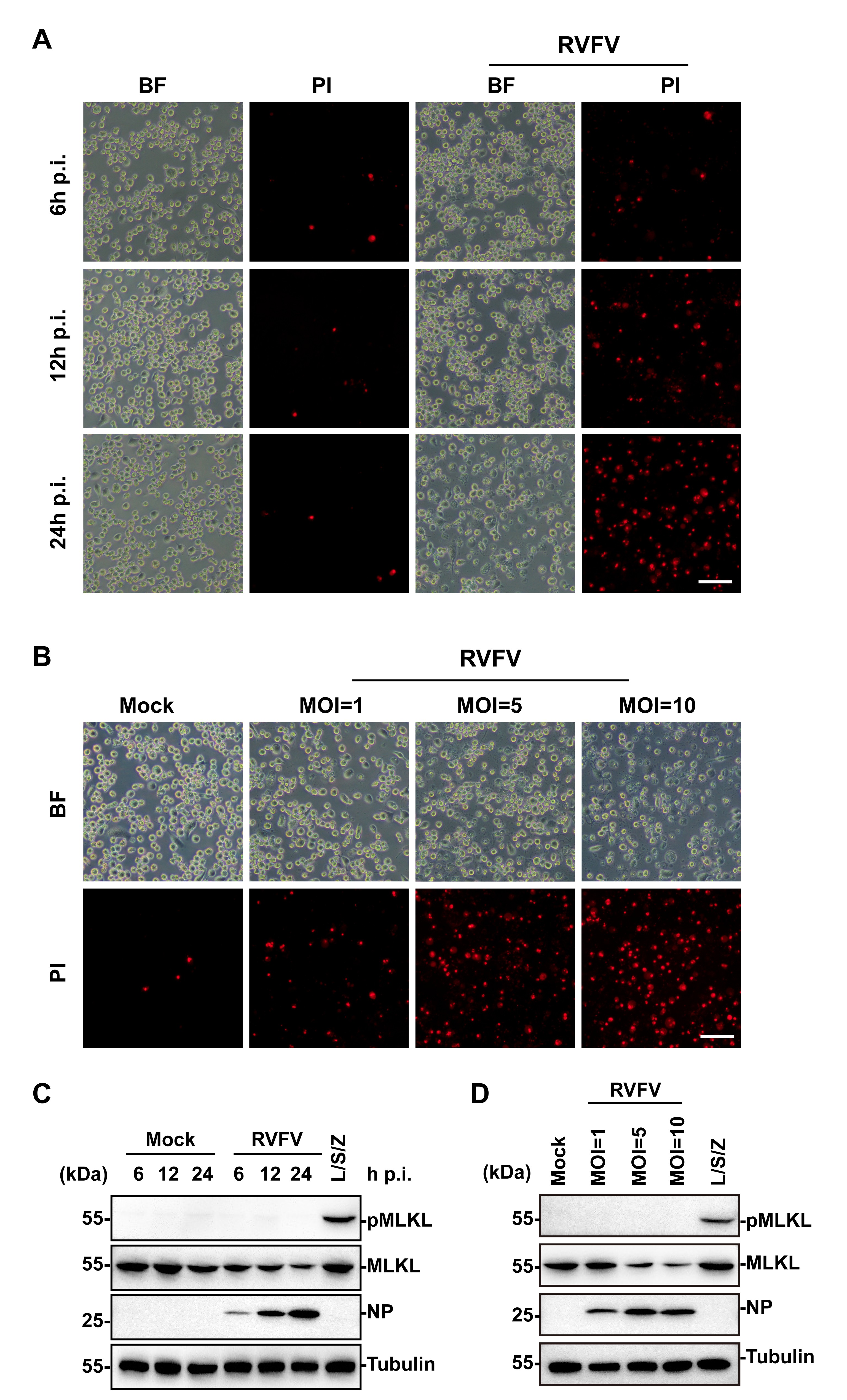

Supplement: S1 Fig — Related to Fig 1. (A and B) Representative images of cell death determined by PI staining in THP-1PMA cells infected with RVFV (MOI = 5) with indicated time (A) and indicated MOI for 24 h (B). BF, bright field. Scale bar, 100 μm. (C and D) Immunoblot analysis of pMLKL in THP-1PMA cells treated with L/S/Z (1 μg/mL LPS, 2.5 μM SM-164, 100 μM Z-VAD) for 6 h, or infected with RVFV (MOI = 5) with indicated time (C) and indicated MOI for 24 h (D). Immunoblot results are representative of three independent experiments. (JPEG) [file ppat.1013113.s001.jpeg]
